# Epidemiological trends of maternal hypertensive disorders of pregnancy at the global, regional, and national levels: a population‐based study

**DOI:** 10.1186/s12884-021-03809-2

**Published:** 2021-05-08

**Authors:** Wei Wang, Xin Xie, Ting Yuan, Yanyan Wang, Fei Zhao, Zhangjian Zhou, Hao Zhang

**Affiliations:** 1 Department of Obstetrics and Gynecology, The First Affiliated Hospital of Xi’an Jiaotong University, 710061 Xi’an, Shaanxi China; 2 Department of Nuclear Medicine, The First Affiliated Hospital of Xi’an Jiaotong University, 710061 Xi’an, Shaanxi China; 3 Department of Oncology, The Second Affiliated Hospital of Xi’an Jiaotong University, 710004 Xi’an, Shaanxi China; 4 Department of Surgical Oncology, The First Affiliated Hospital of Xi’an Jiaotong University, 710061 Xi’an, Shaanxi China; 5 Department of Public Health, Baoji High-tech People’s Hospital, Shaanxi 721000 Baoji, China

**Keywords:** Hypertensive disorders of pregnancy, Burden of disease, Sociodemographic index, Human development index

## Abstract

**Background:**

Relevant studies focusing on epidemiological of profiles hypertensive disorders of pregnancy from global data that report the cause-specific prevalence and trends of hypertensive disorders of pregnancy at global, regional and national levels from 1990 to 2019 by age and sociodemographic index are still limited.

**Methods:**

For hypertensive disorders of pregnancy, point prevalence, annual incidence, and years lived with disability numbers and age standardized rates per 100,000 population were compared at regional and national levels by age and sociodemographic index using data from the global Burden of Disease 2019 Study, covering populations from 204 countries and territories. Estimates are reported with uncertainty intervals to exhibit the changing trends during a specific period.

**Results:**

The incidence of hypertensive disorders of pregnancy increased from 16.30 million to 18.08 million globally, with a total increase of 10.92 % from 1990 to 2019. The age-standardized incidence rate decreased, with an estimated annual percent change of -0.68 (95 % confidence interval [CI] -0.49 to -0.86). The number of deaths due to hypertensive disorders of pregnancy was approximately 27.83 thousand in 2019, representing a 30.05 % decrease from 1990. Based on the incidence and prevalence, the number of deaths and years lived with disability were highest in the group aged 25–29 years, followed by the groups aged 30–34 and 20–24 years, while the lowest estimated incidence rate was observed in the group aged 25–29 years and higher incidence rates were observed in the youngest and oldest groups. Positive associations between incidence rates and the sociodemographic index and human development index were found for all countries and regions in 2019. Age-standardized incidence rates were higher in countries/regions with lower sociodemographic indices and human development indices.

**Conclusions:**

Our study provides a comprehensive overview of the global burden of hypertensive disorders of pregnancy. The death and incidence rates are decreasing in most countries and all regions except for those with low sociodemographic and human development indexes. This difference is mainly due to the increasing attention to prenatal examinations and health education. Further investigations should focus on forecasting the global disease burden of specific hypertensive disorders of pregnancy and modifiable risk factors.

**Supplementary Information:**

The online version contains supplementary material available at 10.1186/s12884-021-03809-2.

## Background

Hypertensive disorders of pregnancy (HDP) is one of the leading causes of maternal and fetal morbidity and mortality worldwide and potentially a critical threat to maternal and infant health [1]. In 2013, the American Congress of Obstetricians and Gynecologists guidelines on hypertensive disorders complicating pregnancy generally classified HDP into four categories: gestational hypertension, preeclampsia/eclampsia, chronic hypertension, and chronic hypertension complicated with preeclampsia/eclampsia [2]. As HDP is considered associated with cardiovascular diseases, the 2020 International Society of Hypertension guidelines continue the framework of the four-category HDP classification scheme, where hemolytic anemia, elevated liver enzymes and low platelet count syndrome (HELLP syndrome) are classified in a separate category [3]. Regardless of how HDP is classified, the importance of blood pressure monitoring during pregnancy is emphasized.

Recent evidence has indicated an increase in the incidence rate of HDP over the past few decades, [7, 8] suggesting that HDP, a sex-specific cardiovascular disease, will become increasingly important in the future [4]. Although the importance of HDP in risk assessment and prevention programs has been recognized, the epidemiology of HDP is not well understood, especially in less developed countries, which may be an obstacle to HDP prevention and proper gestational healthcare strategies. The incidence of HDP ranges from 4 to 25 %, [4, 5] and HDP is one of the three leading causes of maternal morbidity and mortality worldwide. In the past half century, the incidence of preeclampsia and maternal mortality has decreased significantly in developed countries. However, in developing countries, the incidence rates of preeclampsia and maternal mortality are still very high [6]. According to the results of a meta-analysis, the global incidence of preeclampsia is 4.6 % [95 % confidence interval (CI), 2.7–8.2], which varies among different regions, such as 1.0 % in the eastern Mediterranean and 5.6 % in Africa [6]. The incidence of preeclampsia also varies among different countries and regions, and thus the disease burden of HDP is difficult to estimate and HDP prevention is difficult among pregnant women globally [7–11].

In recent years, the burden of HDP has been presented in some review papers based on a few national studies, but no detailed information from all countries has been provided. The overall aim of this study was to determine the epidemiological characteristics of HDP in a population-based cohort. This study presents the modeled global-, regional- and national-level prevalence and incidence rates, mortality rates and years lived with disability (YLDs) of HDP as revealed in the Global Burden of Diseases, Injuries, and Risk Factors Study (GBD) 2019 and analyzes the associations between epidemiological data and socioeconomic factors, such as the sociodemographic index (SDI) and human development index (HDI), to provide comprehensive, comparable and up to date information on the HDP burden.

## Methods

### Data acquisition

The global GBD study includes epidemiological data on the prevalence, incidence, mortality, YLD, disability-adjusted life years (DALYs) and years of life lost (YLLs) for 286 causes of death, 369 diseases and injuries, and 87 risk factors from specific groups in 204 countries and territories by sex, age and year. In the current study, we extracted and analyzed annual data (inclusive dates: 1990 to 2019) on prevalence, incidence, death, DALYs, YLDs, YLLs and the corresponding age-standardized rates (ASRs), as well as risk factors attributable to HDPs from the Global Health Data Exchange (GHDx) database (http://ghdx.healthdata.org/). These data were segmented by country/region and SDI quintile, which reflects the degree of social development and correlates with total fertility, per capita income, and average years of education [12]. Meanwhile, HDI data for 1990 and 2019 were available for 189 countries and regions in the Human Development Report 2019 and Human Development Index Ranking 2019 (http://hdr.undp.org/en/content/2019-human-development-index-ranking). All countries and regions were sorted into five quintiles based on the SDI (http://ghdx.healthdata.org/record/ihme-data/gbd-2019-sociodemographic-index-sdi-1950-2019). Furthermore, factors that might contribute to the progression of HDP were also analyzed, such as micronutrient deficiency. The risk factor was defined as any exposure, behavior, or other factor that was causally related to an increased probability of HDP, while the factor was considered a protective factor if the probability decreased.

### Estimating the number of pregnant women in a population

As HDP occurs during the gestational period, we further estimated the number of pregnant women in a specific year and population. The number of live births in the target year was estimated first (i). Then, the number of pregnancies that ended in stillbirths or miscarriages was estimated (ii) (estimated at 15 % of live births = i × 0.15). Finally, the estimated number of pregnancies expected in a particular year was calculated as i + ii [13]. Data related to the fertility rate are available on the website http://ghdx.healthdata.org/record/ihme-data/gbd-2019-fertility-estimates-1950-2019.

### Statistical analyses

ASRs were analyzed to compare the HDP incidence and mortality trends among different cohorts. DALYs refer to the years lived with disability and years of life lost [14]. Estimated annual percentage changes (EAPCs) indicate ASR trends during a defined period. The specific EAPC was calculated using a generalized linear model considering a Gaussian distribution for the ASR. Based on the assumption of linearity on the log scale, which is equivalent to a constant change assumption, the EAPC was calculated. In addition, world maps and graphs were generated to display the distribution and changing trends in global, regional, and national disease burdens attributable to HDPs. Uncertainty was incorporated by sampling 1,000 draws combining uncertainty from a number of sources, including input data, corrections of measurement errors and estimates of residual nonsampling errors. The uncertainty intervals (UIs) were defined as the 2.5th and 97.5th centiles of the ordered draws. All calculations and figures were performed and generated using EXCEL 2019 (Microsoft Corporation) and R software (version 4.0.0) with “Rcan”, “ggplot2” and other packages, respectively.

## Results

### Global level of HDP

The incidence of HDP increased from 16.30 million [95 % UI 13.56 to 19.42 million] to 18.08 million (95 % UI 15.26 to 21.11 million) globally, with a total increase of 10.92 % from 1990 to 2019. The number of deaths due to HDPs was approximately 27.83 thousand (95 % UI 24.30 to 27.83 thousand) in 2019, with a 30.05 % (95 % UI 28.92–32.71 %) decrease from 1990 to 2019 (Table 1). At the global level, the age-standardized incidence rate (ASIR) decreased from 579 (95 % UI 482 to 689) per 100,000 population in 1990 to 463 (95 % UI 392 to 541) per 100,000 population in 2019, with an EAPC of -0.68 (95 % CI -0.49 to -0.86). Additionally, the EAPC of the age-standardized death rate (ASDR) was − 2.38 (95 % CI 1.67 to -6.27) (Table 1).

**Table 1:** The prevalence of HDP by Global Burden of Disease regions

Characteristics	1990			2019			1990–2019	
	Incidence No. ×10^5^ (95 % UI)	ASIR ×10^2^ (95 % UI)	Death No. ×10^3^ (95 % UI)	Incidence No. ×10^4^ (95 % UI)	ASIR ×10^2^ (95 % UI)	Death No. ×10^3^ (95 % UI)	EAPC of ASIR (95 % CI)	EAPC of ASDR (95 % CI)
Global	162.96 (194.2–135.56)	5.79 (6.89–4.82)	39.79 (43.78–36.11)	180.76 (211.08–152.64)	4.63 (5.41–3.92)	27.83 (31.56–24.3)	-0.68 (-0.49 - -0.86)	-2.38 (1.67 - -6.27)
East Asia	13.9 (17.7–11.03)	1.91 (2.43–1.51)	2.44 (2.99–1.96)	7.34 (9.07–5.96)	0.98 (1.2–0.81)	0.19 (0.23 − 0.14)	-1.52 (-1.13 - -1.9)	-8.17 (4.08 - -18.99)
Southeast Asia	16.63 (20.5–13.37)	6.61 (8.05–5.32)	4.62 (5.5–3.96)	14.79 (17.78–12.06)	4.11 (4.95–3.36)	2.51 (3.01–2.07)	-1.61 (-1.43 - -1.78)	-3.28 (0.38 - -6.8)
Oceania	0.19 (0.23 − 0.15)	5.98 (7.4–4.73)	0.06 (0.07 − 0.04)	0.37 (0.46 − 0.3)	5.46 (6.82–4.36)	0.11 (0.15 − 0.08)	-0.37 (-0.2 - -0.54)	-0.23 (2.79 - -3.17)
Central Asia	0.75 (0.93 − 0.6)	2.07 (2.56–1.67)	0.16 (0.17 − 0.14)	0.86 (1.08–0.69)	1.69 (2.11–1.36)	0.09 (0.11 − 0.08)	-0.15 (0.17 - -0.48)	-2.68 (5.47 - -10.21)
Central Europe	1.12 (1.44–0.86)	1.96 (2.54–1.5)	0.04 (0.04 − 0.03)	0.78 (0.94 − 0.64)	1.61 (1.93–1.32)	0.01 (0.01–0.01)	-0.74 (-0.4 - -1.07)	-4.62 (21.47 - -25.1)
Eastern Europe	3.99 (5.22–3.02)	3.78 (4.96–2.84)	0.15 (0.17 − 0.14)	3.39 (4.28–2.55)	3.67 (4.64–2.79)	0.03 (0.03 − 0.02)	0.86 (1.1–0.63)	-5.87 (11.56 - -20.58)
High-income Asia Pacific	1.66 (2.07–1.3)	1.92 (2.4–1.5)	0.06 (0.07 − 0.05)	1.11 (1.3–0.95)	1.44 (1.69–1.24)	0.01 (0.01–0.01)	-1.71 (-1.39 - -2.03)	-7.65 (22.6 - -30.44)
Australasia	0.34 (0.4 − 0.28)	3.08 (3.66–2.56)	0 (0–0)	0.3 (0.39 − 0.23)	2.17 (2.76–1.67)	0 (0–0)	-1.26 (-0.99 - -1.52)	-2.85 (26.23 - -25.22)
Western Europe	3.48 (4.43–2.71)	1.79 (2.27–1.4)	0.08 (0.09 − 0.08)	3.6 (4.59–2.77)	1.94 (2.48–1.51)	0.02 (0.03 − 0.02)	0.19 (0.51 - -0.12)	-3.91 (26.23 - -26.85)
Southern Latin America	0.95 (1.2–0.74)	3.78 (4.73–2.93)	0.1 (0.11 − 0.09)	1.11 (1.38–0.86)	3.23 (4.03–2.51)	0.06 (0.06 − 0.05)	-0.52 (-0.3 - -0.74)	-2.89 (5.45 - -10.58)
High-income North America	5.41 (6.95–4.17)	3.67 (4.72–2.85)	0.07 (0.08 − 0.07)	5.36 (6.09–4.67)	3.24 (3.68–2.83)	0.09 (0.11 − 0.08)	-0.4 (-0.18 - -0.62)	1.11 (19.5 - -14.45)
Caribbean	0.94 (1.16–0.76)	4.73 (5.76–3.82)	0.21 (0.25 − 0.18)	0.86 (1.04–0.69)	3.55 (4.29–2.88)	0.34 (0.45 − 0.26)	-0.91 (-0.71 - -1.12)	1.63 (5.39 - -2)
Andean Latin America	0.57 (0.65 − 0.51)	2.91 (3.28–2.62)	0.69 (0.79 − 0.6)	0.98 (1.05–0.91)	2.91 (3.13–2.73)	0.36 (0.47 − 0.26)	0.06 (0.29 - -0.17)	-4.49 (-1.72 - -7.17)
Central Latin America	5.61 (6.69–4.7)	6.24 (7.39–5.23)	1.03 (1.1–0.97)	4.87 (5.54–4.27)	3.6 (4.08–3.15)	0.65 (0.8 − 0.52)	-1.46 (-1.25 - -1.66)	-3.33 (1.35 - -7.8)
Tropical Latin America	3.59 (4.45–2.87)	4.17 (5.13–3.36)	0.96 (1.04–0.88)	3.29 (3.85–2.82)	2.77 (3.24–2.38)	0.42 (0.46 − 0.37)	-1.49 (-1.26 - -1.72)	-3.67 (1.89 - -8.93)
North Africa and Middle East	10.9 (13.46–8.75)	6.65 (8.18–5.37)	2.83 (3.21–2.49)	11.72 (14.33–9.46)	3.59 (4.37–2.91)	1.75 (2.28–1.33)	-1.71 (-1.52 - -1.9)	-4.18 (-0.18 - -8.01)
South Asia	43.52 (52.47–35.78)	8.09 (9.69–6.69)	18.74 (21.28–16.21)	38.38 (46.18–31.61)	3.89 (4.67–3.21)	10.34 (12.45–8.34)	-2.7 (-2.54 - -2.87)	-4.14 (-1.28 - -6.91)
Central Sub-Saharan Africa	5.71 (6.75–4.68)	22.42 (26.13–18.56)	1.03 (1.3–0.79)	9.71 (11.54–7.99)	15.18 (17.9–12.59)	1.69 (2.11–1.26)	-1.19 (-1.09 - -1.28)	-0.92 (1.2 - -3.01)
Eastern Sub-Saharan Africa	19.81 (23.16–16.68)	22.46 (25.89–19.11)	3.32 (3.94–2.76)	31.19 (36.29–26.39)	14.96 (17.23–12.71)	4.65 (5.7–3.72)	-1.32 (-1.23 - -1.41)	-1.47 (0.79 - -3.69)
Southern Sub-Saharan Africa	3.24 (3.86–2.67)	11.26 (13.32–9.4)	0.61 (0.7 − 0.54)	3.63 (4.33–3.02)	8.12 (9.68–6.78)	0.46 (0.58 − 0.35)	-1.01 (-0.88 - -1.15)	-1.22 (2.2 - -4.53)
Western Sub-Saharan Africa	20.65 (23.9–17.42)	23.09 (26.31–19.73)	2.57 (3.18–2.06)	37.1 (42.72–31.39)	16.16 (18.42–13.9)	4.07 (5.33–3.13)	-0.97 (-0.87 - -1.06)	-1.39 (1.25 - -3.97)
High-middle SDI	18.37 (22.81–14.64)	2.92 (3.63–2.34)	2.29 (2.54–2.09)	15.41 (18.6–12.45)	2.18 (2.63–1.78)	0.57 (0.64 − 0.51)	-0.57 (-0.3 - -0.83)	-5.25 (4.63 - -14.2)
High SDI	11.15 (13.92–8.86)	2.62 (3.29–2.09)	0.27 (0.3 − 0.25)	10.86 (12.74–9.14)	2.38 (2.8–2.01)	0.15 (0.17 − 0.13)	-0.45 (-0.19 - -0.71)	-1.81 (18.99 - -18.98)
Low-middle SDI	45.51 (54.38–38)	8.15 (9.69–6.82)	17.48 (19.69–15.45)	44.46 (52.08–37.58)	4.65 (5.43–3.93)	9.87 (11.42–8.36)	-1.92 (-1.76 - -2.08)	-3.69 (-0.74 - -6.55)
Low SDI	43.49 (50.63–36.65)	18.04 (20.86–15.26)	9.78 (11.19–8.4)	70.58 (82.16–60.01)	12.65 (14.6–10.77)	13.46 (15.8–11.38)	-1.17 (-1.07 - -1.28)	-1.55 (0.66 - -3.71)
Middle SDI	44.35 (53.47–36.59)	4.69 (5.62–3.88)	9.94 (10.95–9.07)	39.34 (46.14–33.05)	3.14 (3.67–2.64)	3.75 (4.23–3.28)	-1.13 (-0.91 - -1.34)	-4.37 (0.93 - -9.4)

*ASIR* Age-standardized incidence rate, *ASDR *Age-standardized death rate, *EAPC *Estimated annual percentage change, *HDP *Hypertensive disorders of pregnancy, *SDI *Socio-demographic index

### Regional level of HDP

At the regional level, the highest incidence of HDP in 2019 was detected in South Asia [3.84 million (95 % UI 3.16 to 4.62 million)], western sub-Saharan Africa [3.71 million (95 % UI 3.14 to 4.27 million)] and eastern sub-Saharan Africa [3.12 million (95 % UI 2.64 to 3.63 million)]. Conversely, Australasia [30.11 thousand (95 % UI 23.08 to 38.67 thousand)], Oceania [37.06 thousand (95 % UI 29.64 to 46.29 thousand)] and Central Europe [78.06 thousand (95 % UI 64.04 to 94.23 thousand)] had the lowest incidence estimates (Table 1). Western sub-Saharan Africa [1615.93 (95 % UI 1390.04 to 1842.10)], central sub-Saharan Africa [1517.60 (95 % UI 1258.91 to 1790.02)] and eastern sub-Saharan Africa [1495.83 (95 % UI 1271.30 to 1722.85)] had the highest HDP ASIRs, while the lowest values were reported in East Asia [98.03 (95 % UI 80.58 to 120.27)], the high-income Asia Pacific region [144.25 (95 % UI 123.89 to 168.69)] and Central Europe [160.98 (95 % UI 132.18 to 192.85)] (Table 1). Central sub-Saharan Africa [2.80 (95 % UI 2.12 to 3.53)], eastern sub-Saharan Africa [2.40 (95 % UI 1.92 to 2.93)] and western sub-Saharan Africa [1.77 (95 % UI 1.36 to 2.31)] had the highest ASDRs in 2019, while the high-income Asia Pacific region [0.01 (95 % UI 0.01 to 0.01)], Western Europe [0.01 (95 % UI 0.01 to 0.01)] and Australasia [0.01 (95 % UI 0.01 to 0.02)] had the lowest ASDRs (Supplementary Table 1). The EAPCs in the ASIR and ASDR from 1990 to 2019 varied across regions. While all regions showed a decreasing trend for the incidence, Eastern Europe, Western Europe and Andean Latin America displayed potential increasing trends (Table 1). The majority of regions showed decreasing EAPCs in the ASDR trends, except for high-income North America and the Caribbean, which displayed potential increasing trends (Table 1). The age-standardized DALYs, YLLs and YLDs in 2019 are shown in online Supplementary Tables 22, with decreasing trends observed from 1990 to 2019 (Supplementary Fig. 1).

### National level of HDP

**Fig. 1:**
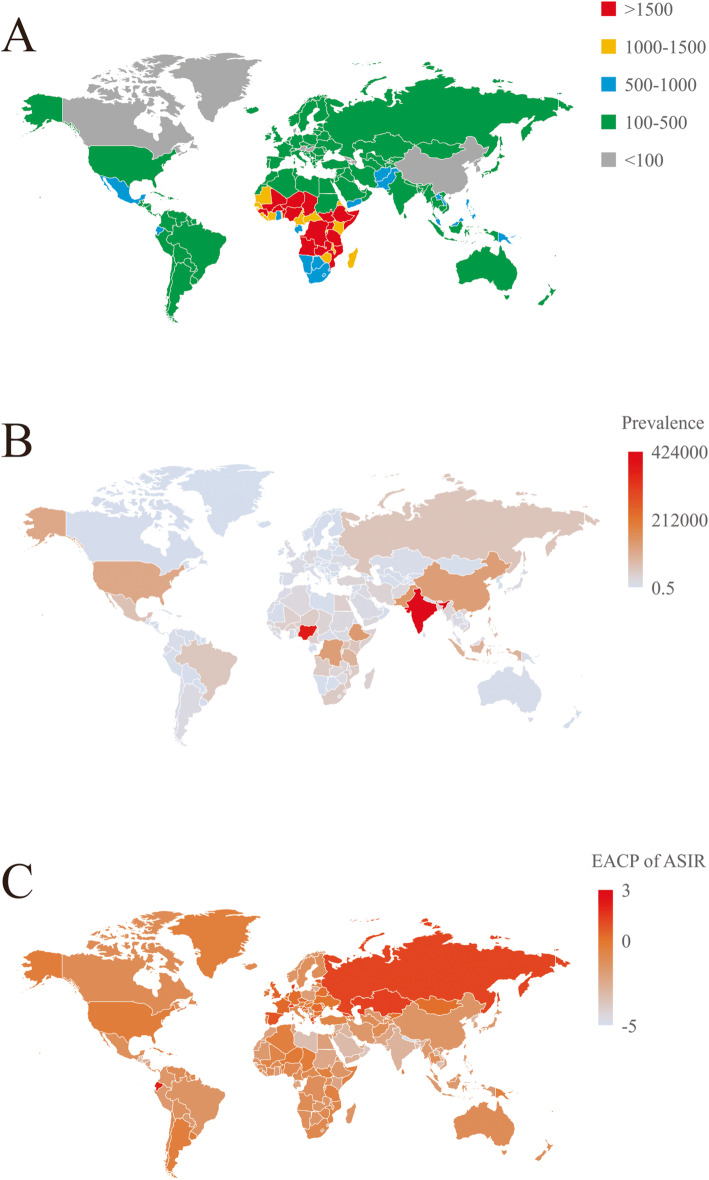
Overview of HDP by country and region for both sexes combined. **a** The prevalence of HDP in 2019. **b** The ASIR of HDP per 100,000 population in 2019. **c** The EAPC in the ASDR of HDP from 1990 to 2019. The map depicted in this figure is our own. ASIR, age-standardized incidence rate; ASDR, age-standardized death rate; EAPC, estimated annual percentage change; HDP, hypertensive disorders of pregnancy

ASIRs per 100,000 population estimates of HDPs in 2019 ranged from 36.34 to 2278.25 among different countries and regions. Chad [2278.25 (95 % UI 1191.82 to 2675.20)], Niger [2256.95 (95 % UI 1883.97 to 2644.81)] and Somalia [2251.26 (95 % UI 1893.54 to 2597.09)] were the countries with the highest ASIR estimates in 2019. Korea [36.34 (95 % UI 29.61 to 45.73)], Canada [49.13 (95 % UI 41.84 to 57.00)] and Luxembourg [58.27 (95 % UI 45.34 to 74.77)] had the lowest estimates (Fig. 1a and online Supplementary Table 3). All age prevalence number estimates in 2019 differed substantially among countries, with the lowest values observed in Niue Island [0.60 (95 % UI 0.36 to 0.89)], Tokelau [0.81 (95 % UI 0.48 to 1.21)], and Tuvalu [4.14 (95 % UI 2.50 to 6.24)] and the highest values observed in India [423997.43 (95 % UI 266164.82 to 623364.13)], Nigeria [367632.29 (95 % UI 234623.34 to 518038.38)], and Pakistan [143309.61 (95 % UI 89458.08 to 209804.74)] (data shown in Fig. 1b and online Supplementary Table 4).

The EAPC in ASIR estimates from 1990 to 2019 differed substantially among countries. One hundred seventy-four countries and regions displayed decreasing trends, while 30 countries and regions exhibited increasing trends. Nepal [-4.64 (95 % CI -4.83 to -4.45)], Qatar [-3.97 (95 % CI -4.19 to -3.75)] and Oman [-3.68 (95 % CI -3.86 to -3.50)] showed the greatest decreasing trends. Ecuador [2.09 (95 % CI 1.90 to 2.28)], Switzerland [1.55 (95 % CI 1.14 to 1.96)] and Denmark [1.52 (95 % CI 0.98 to 2.06)] showed the greatest increasing trends (Fig. 1c and online Supplementary Table 5).

### Age distribution of HDP

**Fig. 2:**
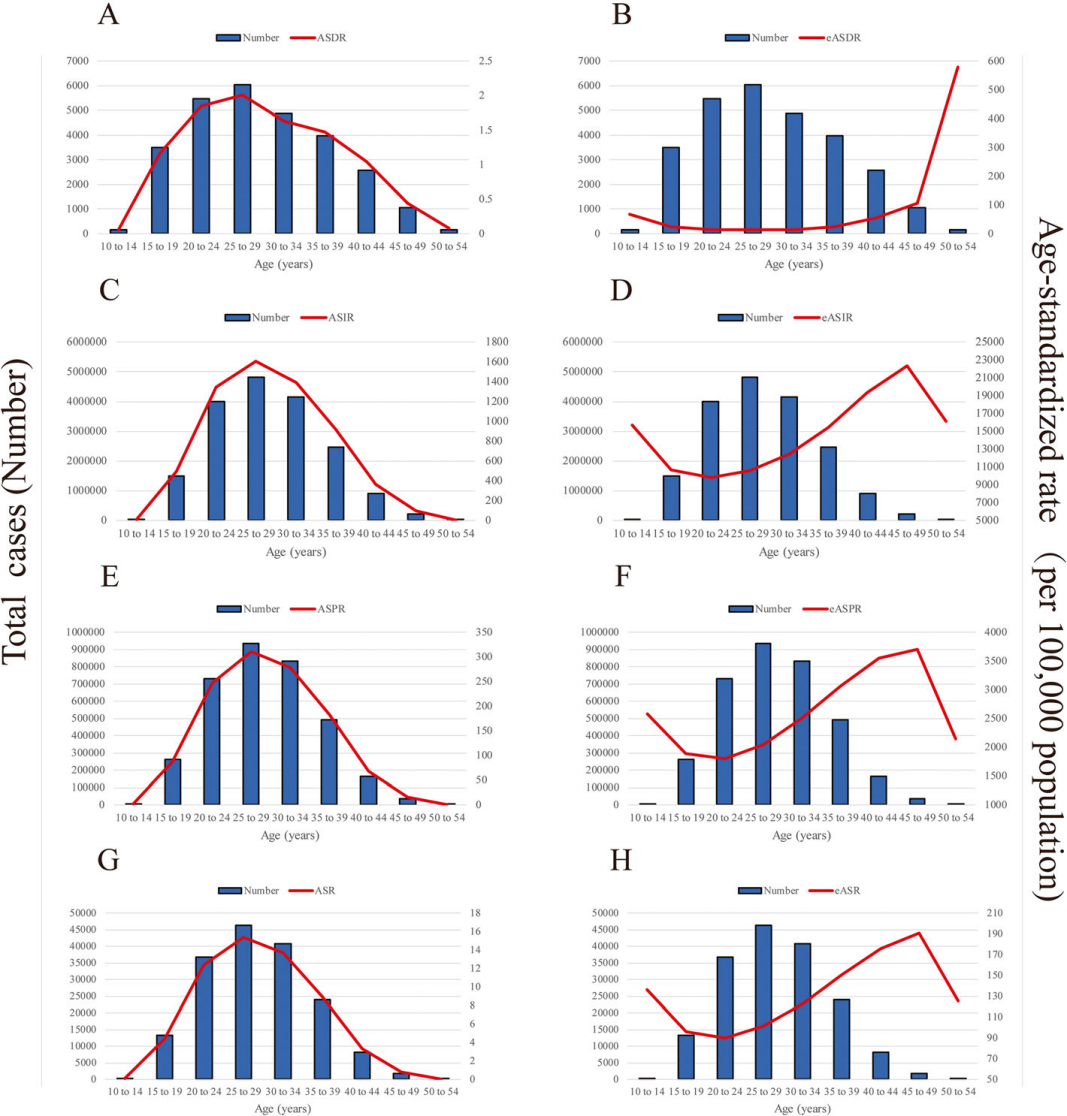
Global number of cases and ASRs per 100,000 population stratified by age in 2017. **a**The global number of deaths and ASDRs. **b** The global number of deaths and eASDR. **c** The global number of incident cases and ASIR. **d** The global number of incident cases and eASIR. **e** The global number of prevalence cases and ASPR. **f** The global number of prevalent cases and eASPR. **g** The global number of YLDs and ASR of YLDs. **h** The global number of YLDs and eASR of YLDs. ASIR, age-standardized incidence rate; eASIR, estimated age-standardized incidence rate according to the number of pregnant women; ASDR, age-standardized death rate; eASDR, estimated age-standardized death rate according to the number of pregnant women; ASPR, age-standardized prevalence rate; eASPR, estimated age-standardized prevalence rate according to the number of pregnant women; ASR, age-standardized rate; eASR, estimated age-standardized rate according to the number of pregnant women; YLDs, years lived with disability

From 1990 to 2019, the global HDP incidence, prevalence, death and YLDs were highest in populations aged 25–29 years, followed by populations aged 30–34 and 20–24 years (Fig. 2). The trend in the HDP incidence was similar to the normal distribution, with the lowest values observed in women aged 10–14 and 55–59 years. Similar trends were also observed in the prevalence, death and YLD data. However, as the target population of HDP is pregnant women, the number of pregnant women was further estimated according to the fertility rate and the incidence rate of HDP based on pregnant women was calculated; the lowest incidence rate was observed in the group aged 25–29 years but higher rates were observed in the youngest and oldest age groups (Fig. 2). Similar trends were also observed in prevalence, death and YLD data.

### Burden of HDP in regions stratified by SDI and HDI

**Fig. 3:**
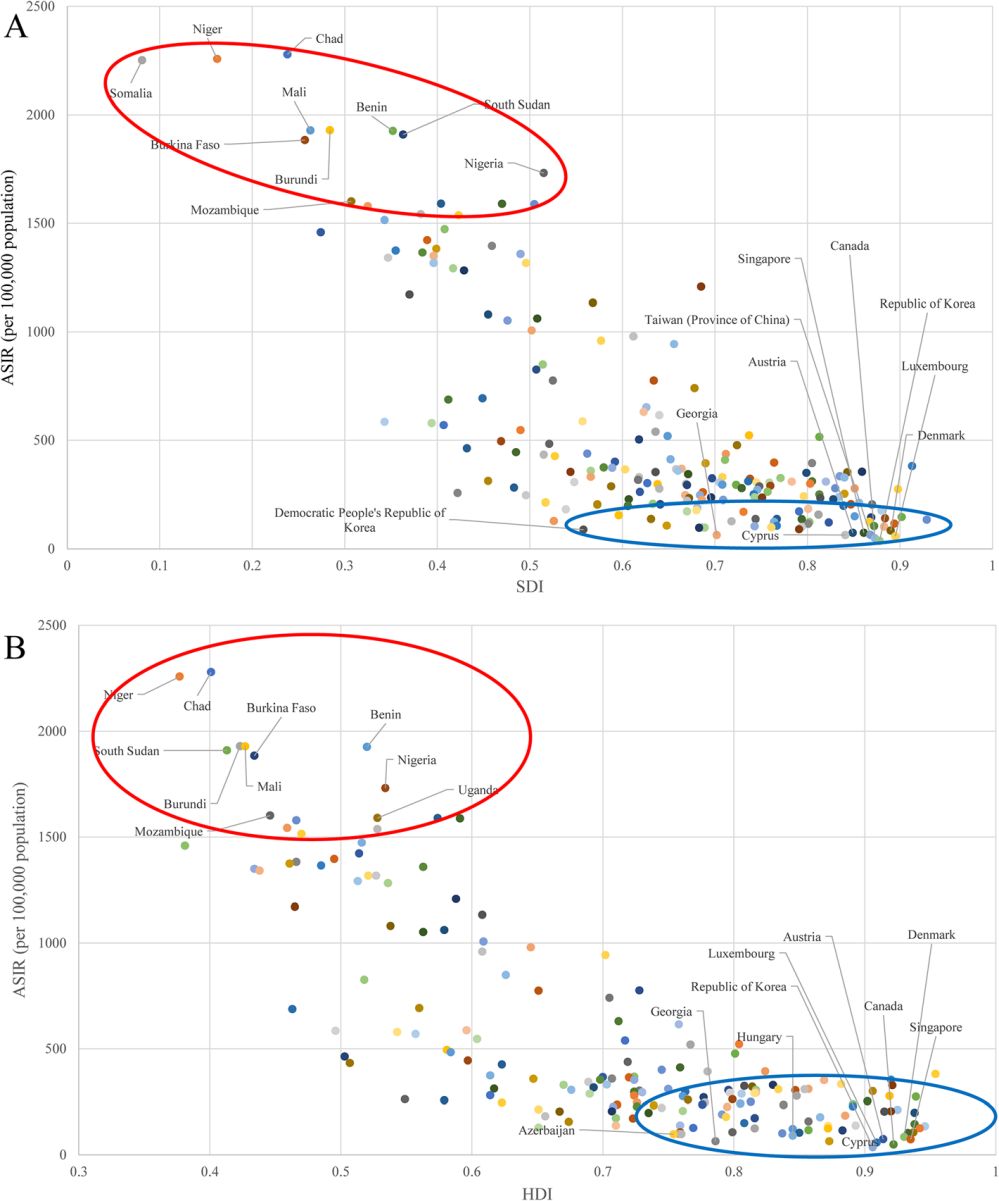
ASIRs of HDP by country and region and (**a**) SDIs (with 204 countries and regions) and (**b**) HDIs (with 187 countries and regions) in 2019. Each point shows the observed ASIR for a specified country or region in 2019. The red circle includes the 10 countries or regions with the highest ASIRs. The blue circle includes the 10 countries or regions with the lowest ASIRs. ASIR, age-standardized incidence rate; HDP, hypertensive disorders of pregnancy; HDI, human development index; SDI, sociodemographic index

Generally, a positive association between the ASIR and SDI was found for all countries and regions in 2019. The ASIRs were higher in countries/regions with lower SDIs (Fig. 3a and online Supplementary Table 6). The SDIs for Chad, Niger, and Somalia, the three countries with the highest incidence rates, were 0.24, 0.16, and 0.08, respectively. The SDIs for Korea, Canada, and Luxembourg, the three countries with the lowest incidence rates, were 0.88, 0.87, and 0.90, respectively. The incident HDP case number increased globally, which was mainly attributed to the increasing trend of low SDI areas (Supplementary Fig. 2).

Similarly, a positive association between the ASIR and HDI was also observed for all countries and regions in 2019. The ASIRs were higher in countries/regions with lower HDIs (Fig. 3b and online Supplementary Table 7). The HDIs of Chad, Niger, and Burundi, the three countries with the highest incidence rates, were 0.401, 0.377, and 0.423, respectively. The SDIs of Korea, Canada, and Luxembourg, the three countries with the lowest incidence rates, were 0.906, 0.922, and 0.909, respectively.

### Risk factors for the HDP burden

The global HDP ASDR attributable to all risk factors decreased from 0.34 (95 % UI 0.12 to 0.54) to 0.15 (95 % UI 0.05 to 0.25) per 100,000 population between 1990 and 2019. Iron deficiency was recognized as a risk factor for HDP progression. Unfortunately, data on several factors were limited in the GBD study, such as body mass index, maternal age and preexisting diseases, which impeded the assessment of the HDP risk.

## Discussion

As HDP is considered one of the major causes of maternal mortality worldwide, epidemiological surveillance of HDP is critical to gestational healthcare [15]. To the best of our knowledge, this study is the first to analyze the trends in the HDP incidence at the global, regional and national levels and to examine the associations of the ASR of and EAPC in HDP with SDI and HDI in different countries or regions. From 1990 to 2019, the ASIR of HDPs decreased by an annual average of 0.68 %, while the total number of incident cases increased by 10.92 %. This pattern appears to suggest a major public health problem, as the ASIR has not substantially improved. The number of incident cases increased, probably due to population growth and multiple gestations. Notably, although the number of incident cases of HDP increased from 1990 to 2019, the incidence rate showed a decreasing trend, which was related to the increase in the global population. Moreover, both the number of deaths due to and ASDR of HDPs showed decreasing trends, which may be due to the advances medical interventions and gradually increasing attention to HDPs by clinicians worldwide [7].

Socioeconomic status is associated with several diseases, such as neurological and immunorelated diseases [16]. Obstetricians have known for many years that women with HDP generally have low total family incomes because the mortality rate of HDP is inversely correlated with average family income and maternal deaths from HDP/eclampsia are highest in areas of poverty [17]. According to a previous study, the incidence of HDP varies according to family income. When grouped by income, the highest incidence rates of preeclampsia are observed in upper middle-income countries, whereas eclampsia appears to be more frequent in lower middle-income countries [6]. In our study, both the incident case numbers and rates were highest in the low-SDI areas. At the national level, HDP incidence also showed a decreasing trend as SDI increased. A similar trend was also observed between ASIR and HDI, indicating important roles of economic support in gestational healthcare for both pregnant women and fetuses.

Previous studies reported that the risk of preeclampsia increased in an approximately linear manner with maternal age and that this pattern was similar in nulliparous and multiparous women [18]. Luo et al. identified the incidence rates of pregnancy complications in nulliparous and multiparous Chinese women of advanced maternal age using a community-based prospective cohort. The findings suggested that women of advanced maternal age should be regarded as at high risk for pregnancy complications, including HDP [19]. A large cohort of Japanese women with singleton pregnancies also indicated that advanced maternal age was a risk factor for HDP [10]. Surprisingly, according to GBD database information, the incidence rate of HDP was highest in the group aged 25–29 years in our study, because the population included in the calculation comprised all women in that age group, not only pregnant women. This phenomenon was also observed in the GBD-based “Maternal Health Report” (https://maternalhealthatlas.org/) [20]. However, when using the fertility rate and other information to estimate the number of pregnant women during a period of time and then calculating the incidence of HDP based only on pregnant women, the results showed the lowest incidence in the group aged 25–29 years, similar to the results of previous studies. In general, HDP is more likely to occur at the extremes of reproductive age. Women of advanced maternal age are expected to have an increased rate of HDP because of risk factors such as obesity and diabetes [21] and are more likely to develop atherosclerosis, which affects the small arteries, leading to hypertension [17, 22]. Furthermore, teenage pregnancy is largely associated with adverse pregnancy outcomes such as preterm delivery, preeclampsia and low birth weight [23]. These outcomes might be related to poverty, inadequate nutrition, poor health before pregnancy, marital status, or a low education level, which lead to limited healthcare knowledge among teenagers [24].

In the GBD database, 87 risk factors were included, as they might cause 369 diseases or injuries globally, including environmental and occupational risks, such as air pollution; behavioral risks, such as iron deficiency; and metabolic risks, such as a high body mass index. In our study, only iron deficiency from the behavioral risk category was included as a risk factor for HDP in the population, while information on other risk factors was limited. However, previous studies have shown correlations between these “missing” factors and HDP progression. As a metabolic factor, women’s blood lipid levels change significantly in early pregnancy. Researchers have found that approximately 20–40 % of patients with preeclampsia have foam cell deposits in the subcutaneous fat and intraarterial cellulose and platelet aggregation [25]. Data from a population study show significantly higher fasting triglyceride, total cholesterol and low-density lipoprotein-c levels in the HDP group than those in the healthy control group, and early detection of these parameters may help to better manage HDP [26]. In addition, ambient air pollution, an important environmental risk factor, is a complex mixture composed of gases, aerosols, particulate matter (such as PM_10_ and PM_2.5_). The GBD results show that in 2019, only atmospheric PM_2.5_ pollution directly or indirectly caused 6.67 million deaths and 213.28 million DALYs. Recently, some studies have discussed the possible association between air pollution and HDP [27, 28]. A study recruited 22,041 pregnant women and found that exposure to high levels of air pollution in the early stage of pregnancy and throughout pregnancy were associated with HDP [particulate matter (PM_2.5_): odds ratio (OR) = 1.33, 95 % CI: 1.18–1.49, NO_2_: OR = 1.21, 95 % CI: 1.09–1.35, SO_2_: OR = 1.13, 95 % CI: 1.01–1.25, CO: OR = 1.12, 95 % CI: 1.03–1.22] [29]. Studies by Mobasher et al. suggested that only PM_2.5_ exposure in early pregnancy (OR = 9.10, 95 % CI: 3.33–24.6) and CO exposure in the early and middle stages of pregnancy (early pregnancy: OR = 4.96, 95 % CI: 1.85–13.31, second trimester: OR = 2.05, 95 % CI: 1.22–3.46) increase the risk of HDP [30]. Although indicators related to lipid metabolism and air pollution are included in the GBD database, relevant data were not available for the HDP analysis. Thus, we were only able to analyze behavior-related risk factors in this study, which was a limitation, and further investigation is also needed.

Our study found a decreasing trend in HDP due to iron deficiency from 1990 to 2019, which may be related to an increasing number of obstetricians prescribing iron supplements during pregnancy. However, some controversy still exists regarding the relationship between iron supplementation during pregnancy and the occurrence of HDP. Maintaining optimal iron levels is a delicate balance, with high levels associated with toxicity. One study found that pregnant women who subsequently developed HDP had lower serum iron levels in the first trimester than those who did not develop HDP. However, other studies showed different trends [31]. Another study indicated higher serum iron levels measured at 20 weeks of gestation in women with preeclampsia but not gestational hypertension than in women with normotensive pregnancies [32]. Differences in these findings are potentially explained by the use of different methodologies and gestational times of serum sample collection [31]. Thus, adequate iron intake may be a concern in pregnancy [33].

## Conclusions

Our study provides a comprehensive overview of the global HDP burden. Although variations were observed between regions and countries in the prevalence of, incidence of and mortality due to HDP, the burden of HDP is decreasing in most countries and in all regions. This decrease is mainly because prenatal examinations and health education have received increasing attention. The prevalence of HDP was associated with the sociodemographic index and human development index. Further investigations should focus on forecasting the global disease burden of specific classifications of HDP and modifiable risk factors.

## Supplementary Information

Additional file 1: **Supplementary Figure 1.** The changes in ASRs of DALYs, YLDs and YLLs in different global regions from 1990 to 2019. ASR, age-standardized rate; DALYs, disability-adjusted life years; HDP, hypertensive disorders of pregnancy; YLDs, years lived with disability; YLLs, years of life lost.

Additional file 2: **Supplementary Figure 2.** The incident number of HDP cases in the different SDI quintiles. HDP, hypertensive disorders of pregnancy; SDI, sociodemographic index.

Additional file 3: **Supplementary Table 1.** The age-standardized death rate of HDP in 2019.

Additional file 4: **Supplementary Table 2.** The ASRs of DALYs, YLDs and YLLs of HDP in 2019.

Additional file 5: **Supplementary Table 3.** ASIR of HDP in different countries and regions in 2019.

Additional file 6: **Supplementary Table 4.**The prevalence of HDP in different countries and regions in 2019.

Additional file 7: **Supplementary Table 5.**The EACP of the ASIR of HDP in different countries and regions from 1990 to 2019.

Additional file 8: **Supplementary Table 6.** The relationship between the ASIR and SDI.

Additional file 9: **Supplementary Table 7.** The relationship between the ASIR and HDI.

## Data Availability

The datasets supporting the conclusions of this article are included within the article (and its additional files) and in the open accessed GHDx database (http://ghdx.healthdata.org/).

## Abbreviations

ASR: Age-standardized rate
ASDR: Age-standardized deaths rate
ASIR: Age-standardized incidence rate
CI: Confidence interval
DALY: Disability-adjusted life year
EAPC: Estimated annual percentage change
GBD: Global Burden of Diseases, Injuries, and Risk Factors Study
GHDx: Global Health Data Exchange
HDP: Hypertensive disorders of pregnancy
HDI: Human development index
HELLP: Hemolytic anemia, elevated liver function and low platelet count syndrome
OR: Odds ratio
PM: Particulate matter
SDI: Sociodemographic index
UI: Uncertainty intervals
YLDs: Years lived with disability
YLLs: Years of life lost
