# Supplementary figures and images for: Epidemiological trends of maternal hypertensive disorders of pregnancy at the global, regional, and national levels: a population‐based study
Source: BMC Pregnancy Childbirth. 2021 May 8;21:364. doi: 10.1186/s12884-021-03809-2 (PMC8106862; doi:10.1186/s12884-021-03809-2)

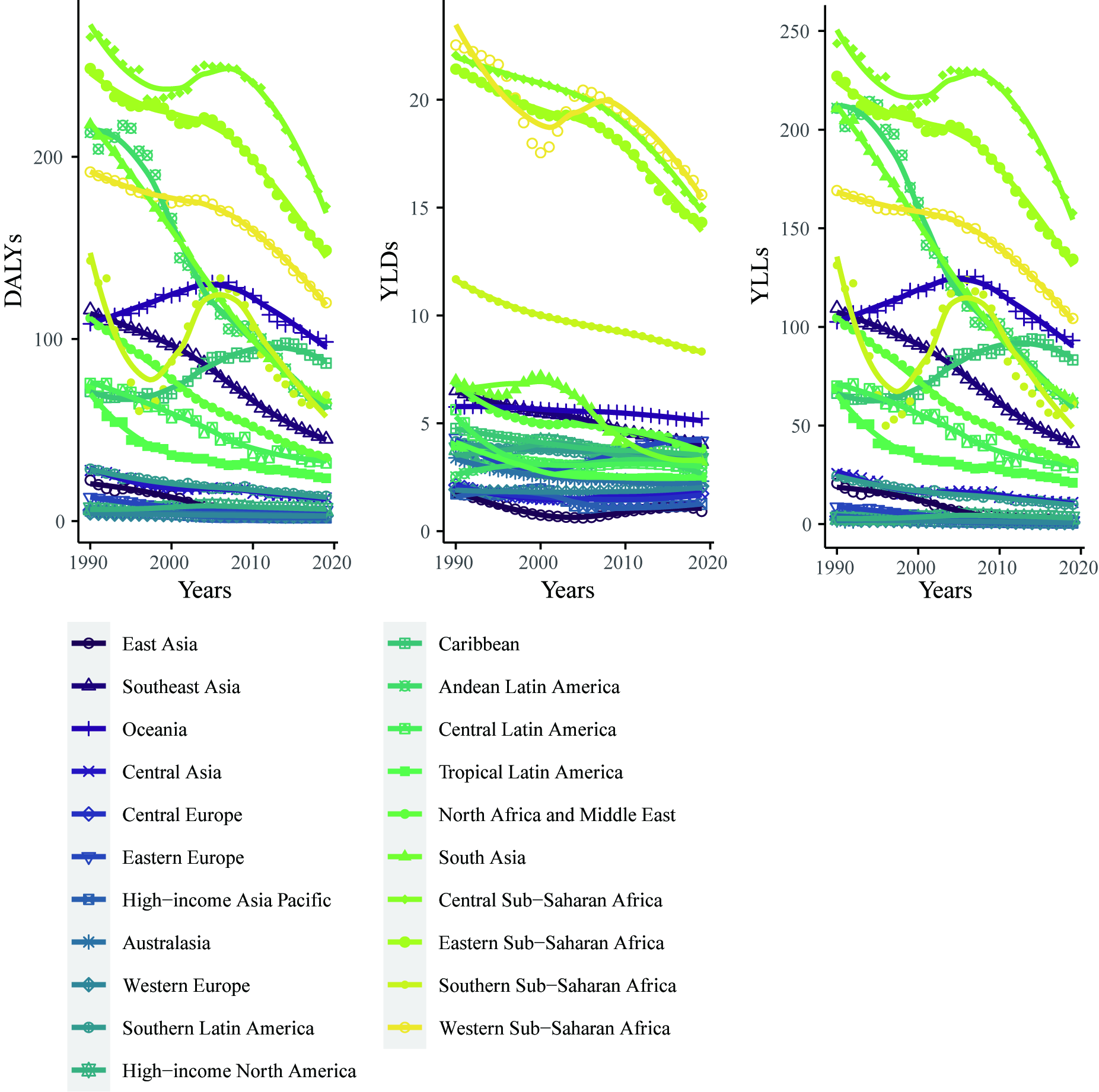

Supplement: Supplementary file 1 — Supplementary Figure 1. The changes in ASRs of DALYs, YLDs and YLLs in different global regions from 1990 to 2019. ASR, age-standardized rate; DALYs, disability-adjusted life years; HDP, hypertensive disorders of pregnancy; YLDs, years lived with disability; YLLs, years of life lost. [file 12884_2021_3809_MOESM1_ESM.tif]

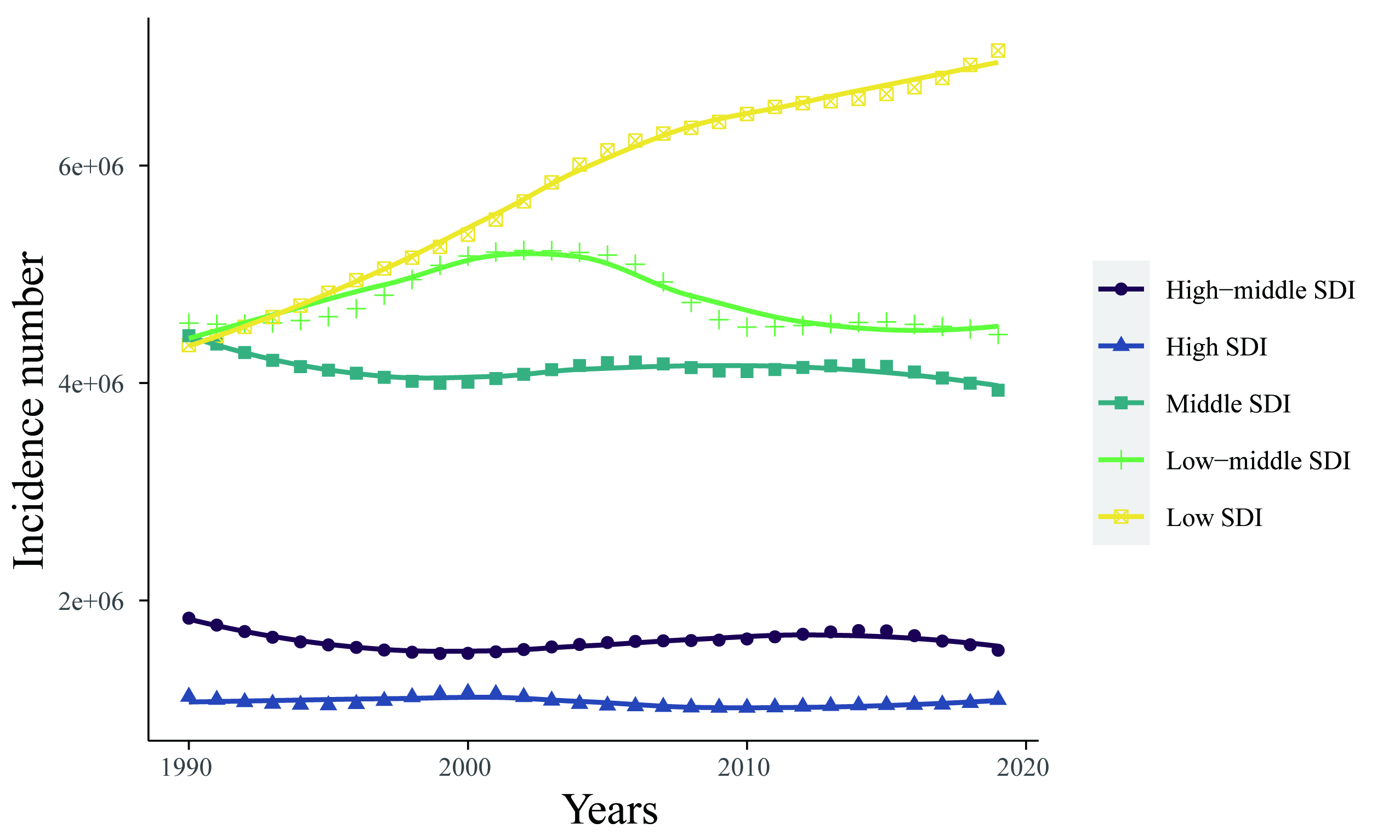

Supplement: Supplementary file 2 — Supplementary Figure 2. The incident number of HDP cases in the different SDI quintiles. HDP, hypertensive disorders of pregnancy; SDI, sociodemographic index. [file 12884_2021_3809_MOESM2_ESM.tif]
