# Supplementary material for: Epidemiological trends of maternal hypertensive disorders of pregnancy at the global, regional, and national levels: a population‐based study
Source: BMC Pregnancy Childbirth. 2021 May 8;21:364. doi: 10.1186/s12884-021-03809-2 (PMC8106862; doi:10.1186/s12884-021-03809-2)
Supplement: Supplementary file 3 — Supplementary Table 1. The age-standardized death rate of HDP in 2019. [file 12884_2021_3809_MOESM3_ESM.docx]

Supplementary Table 1 The age-standardized death rate of HDP in 2019

| Characteristics | ASDR | 95% UI upper | 95% UI lower |
| --- | --- | --- | --- |
| East Asia | 0.0262 | 0.032555 | 0.020398 |
| Southeast Asia | 0.696585 | 0.835594 | 0.575893 |
| Oceania | 1.605334 | 2.178363 | 1.184166 |
| Central Asia | 0.181249 | 0.215244 | 0.155144 |
| Central Europe | 0.01756 | 0.020872 | 0.014646 |
| Eastern Europe | 0.028217 | 0.03453 | 0.022737 |
| High-income Asia Pacific | 0.009251 | 0.010648 | 0.007973 |
| Australasia | 0.01385 | 0.016407 | 0.011566 |
| Western Europe | 0.013109 | 0.014224 | 0.011976 |
| Southern Latin America | 0.166526 | 0.187536 | 0.145914 |
| High-income North America | 0.054269 | 0.063029 | 0.046157 |
| Caribbean | 1.412524 | 1.837721 | 1.067339 |
| Andean Latin America | 1.063691 | 1.395142 | 0.775253 |
| Central Latin America | 0.476384 | 0.590254 | 0.382295 |
| Tropical Latin America | 0.347601 | 0.382704 | 0.312454 |
| North Africa and Middle East | 0.543167 | 0.708365 | 0.412718 |
| South Asia | 1.057648 | 1.276345 | 0.853025 |
| Central Sub-Saharan Africa | 2.80106 | 3.531024 | 2.105804 |
| Eastern Sub-Saharan Africa | 2.399134 | 2.934813 | 1.915773 |
| Southern Sub-Saharan Africa | 1.049295 | 1.332986 | 0.79837 |
| Western Sub-Saharan Africa | 1.765111 | 2.305069 | 1.35928 |

ASDR, age-standardized death rate; HDP, hypertensive disorders of pregnancy.
