# Supplementary material for: Epidemiological trends of maternal hypertensive disorders of pregnancy at the global, regional, and national levels: a population‐based study
Source: BMC Pregnancy Childbirth. 2021 May 8;21:364. doi: 10.1186/s12884-021-03809-2 (PMC8106862; doi:10.1186/s12884-021-03809-2)
Supplement: Supplementary file 4 — Supplementary Table 2. The ASRs of DALYs, YLDs and YLLs of HDP in 2019. [file 12884_2021_3809_MOESM4_ESM.docx]

Supplementary Table 2 The ASRs of DALYs, YLDs and YLLs of HDP in 2019

| Characteristics | DALYs | | YLDs | | YLLs | |
| --- | --- | --- | --- | --- | --- | --- |
|  | Rate | 95% UI | Rate | 95% UI | Rate | 95% UI |
| East Asia | 2.48 | 1.95 - 3.15 | 0.92 | 0.49 - 1.49 | 1.56 | 1.22 - 1.93 |
| Southeast Asia | 45 | 37.82 - 53.24 | 4.03 | 2.14 - 6.67 | 40.97 | 34.02 - 49.34 |
| Oceania | 98.34 | 73.95 - 131.28 | 5.21 | 2.71 - 8.8 | 93.13 | 69.04 - 125.74 |
| Central Asia | 12.68 | 10.71 - 14.95 | 1.73 | 0.92 - 2.85 | 10.94 | 9.35 - 13.03 |
| Central Europe | 2.8 | 1.93 - 3.95 | 1.74 | 0.92 - 2.87 | 1.07 | 0.89 - 1.27 |
| Eastern Europe | 5.89 | 3.81 - 8.79 | 4.17 | 2.16 - 7.04 | 1.71 | 1.38 - 2.09 |
| High-income Asia Pacific | 1.78 | 1.22 - 2.57 | 1.24 | 0.68 - 2.01 | 0.53 | 0.46 - 0.62 |
| Australasia | 3.13 | 1.97 - 4.72 | 2.31 | 1.17 - 3.88 | 0.82 | 0.68 - 0.96 |
| Western Europe | 2.85 | 1.83 - 4.3 | 2.09 | 1.07 - 3.53 | 0.77 | 0.7 - 0.83 |
| Southern Latin America | 13.62 | 11.44 - 16.41 | 3.55 | 1.87 - 5.96 | 10.07 | 8.79 - 11.38 |
| High-income North America | 6.46 | 4.85 - 8.55 | 3.33 | 1.8 - 5.43 | 3.13 | 2.67 - 3.64 |
| Caribbean | 86.71 | 66.61 - 110.72 | 3.47 | 1.84 - 5.72 | 83.24 | 63.35 - 107.59 |
| Andean Latin America | 64.25 | 47.7 - 83.7 | 2.73 | 1.52 - 4.35 | 61.52 | 45.09 - 80.78 |
| Central Latin America | 31.83 | 26.07 - 38.87 | 3.13 | 1.72 - 5.01 | 28.69 | 23.07 - 35.4 |
| Tropical Latin America | 23.47 | 20.85 - 26.25 | 2.51 | 1.38 - 4.09 | 20.96 | 18.77 - 23.14 |
| North Africa and Middle East | 34.7 | 27.05 - 44.37 | 3.82 | 2.02 - 6.29 | 30.88 | 23.57 - 40.48 |
| South Asia | 66.43 | 54.17 - 79.03 | 3.22 | 1.71 - 5.23 | 63.2 | 50.9 - 75.92 |
| Central Sub-Saharan Africa | 172.81 | 134.01 - 215 | 15.05 | 8.04 - 24.26 | 157.76 | 118.17 - 197.72 |
| Eastern Sub-Saharan Africa | 148.63 | 120.1 - 180.81 | 14.32 | 7.82 - 22.97 | 134.32 | 107.56 - 164.82 |
| Southern Sub-Saharan Africa | 69.15 | 53.44 - 87.5 | 8.33 | 4.42 - 13.45 | 60.82 | 46.06 - 77.91 |
| Western Sub-Saharan Africa | 119.89 | 93.57 - 152.71 | 15.6 | 8.61 - 24.84 | 104.29 | 80.18 - 136.22 |

ASR, age-standardized rate; DALY, disability-adjusted life years; HDP, hypertensive disorders of pregnancy;

YLD, years lived with disability; YLL, years of life lost.
