# Supplementary material for: Epidemiological trends of maternal hypertensive disorders of pregnancy at the global, regional, and national levels: a population‐based study
Source: BMC Pregnancy Childbirth. 2021 May 8;21:364. doi: 10.1186/s12884-021-03809-2 (PMC8106862; doi:10.1186/s12884-021-03809-2)
Supplement: Supplementary file 5 — Supplementary Table 3. ASIR of HDP in different countries and regions in 2019. [file 12884_2021_3809_MOESM5_ESM.docx]

Supplementary Table 3 ASIR of HDP at different countries and regions in 2019

| Countries and regions | ASIR | 95% UI upper | 95% UI lower |
| --- | --- | --- | --- |
| Afghanistan | 584.4843 | 736.2291 | 461.5937 |
| Albania | 190.2419 | 248.2802 | 141.382 |
| Algeria | 411.3353 | 529.8306 | 318.3739 |
| American Samoa | 436.4702 | 541.1998 | 341.0609 |
| Andorra | 116.427 | 153.8939 | 85.16181 |
| Angola | 1589.578 | 1899.668 | 1302.526 |
| Antigua and Barbuda | 236.2492 | 294.8105 | 188.3942 |
| Argentina | 329.2512 | 415.9226 | 253.8699 |
| Armenia | 97.97534 | 125.0265 | 76.47961 |
| Australia | 198.5863 | 260.5149 | 146.9007 |
| Austria | 73.82165 | 76.32711 | 71.42426 |
| Azerbaijan | 97.48303 | 123.5277 | 77.44915 |
| Bahamas | 241.3242 | 296.9024 | 191.7219 |
| Bahrain | 234.5714 | 301.1549 | 182.3561 |
| Bangladesh | 280.8735 | 350.077 | 222.7589 |
| Barbados | 250.1805 | 309.7235 | 199.8187 |
| Belarus | 305.4828 | 402.8243 | 224.3147 |
| Belgium | 277.3669 | 368.1244 | 200.2292 |
| Belize | 365.1282 | 453.4774 | 287.9221 |
| Benin | 1926.304 | 2229.036 | 1640.953 |
| Bermuda | 249.9429 | 315.4645 | 194.5299 |
| Bhutan | 311.656 | 392.7791 | 244.6671 |
| Bolivia (Plurinational State of) | 330.763 | 401.3425 | 267.173 |
| Bosnia and Herzegovina | 127.2031 | 168.1859 | 94.23622 |
| Botswana | 775.7431 | 920.3837 | 637.8801 |
| Brazil | 276.5729 | 322.7275 | 238.0347 |
| Brunei Darussalam | 121.6909 | 153.9651 | 98.07386 |
| Bulgaria | 124.6706 | 145.7755 | 106.4243 |
| Burkina Faso | 1884.186 | 2199.525 | 1580.93 |
| Burundi | 1929.532 | 2223.959 | 1609.221 |
| Cabo Verde | 775.2664 | 906.1806 | 650.4194 |
| Cambodia | 494.8463 | 623.6401 | 388.365 |
| Cameroon | 1358.188 | 1576.35 | 1146.873 |
| Canada | 49.13088 | 57.00088 | 41.83726 |
| Central African Republic | 1458.542 | 1749.03 | 1199.057 |
| Chad | 2278.252 | 2675.201 | 1911.821 |
| Chile | 304.7571 | 383.9893 | 232.0676 |
| China | 98.7178 | 121.2243 | 80.97227 |
| Colombia | 206.6408 | 242.0396 | 176.9244 |
| Comoros | 1080.661 | 1238.653 | 916.5619 |
| Congo | 1133.299 | 1343.052 | 935.4177 |
| Cook Islands | 396.5173 | 491.5775 | 310.3259 |
| Costa Rica | 178.6249 | 210.0858 | 152.1484 |
| Croatia | 101.0737 | 106.1053 | 96.83153 |
| Cuba | 246.3622 | 309.682 | 194.0272 |
| Cyprus | 63.98918 | 84.57873 | 47.28773 |
| Czechia | 227.6891 | 297.529 | 165.3872 |
| Cote d'Ivoire | 1473.049 | 1726.349 | 1242.296 |
| Democratic People's Republic of Korea | 88.52014 | 115.3592 | 70.02241 |
| Democratic Republic of the Congo | 1542.653 | 1822.36 | 1257.78 |
| Denmark | 84.63659 | 111.4499 | 64.10314 |
| Djibouti | 1395.901 | 1599.605 | 1193.218 |
| Dominica | 278.7147 | 344.5973 | 218.3856 |
| Dominican Republic | 400.2348 | 500.9488 | 310.5924 |
| Ecuador | 615.1275 | 621.9633 | 608.3171 |
| Egypt | 366.6369 | 460.4675 | 288.2453 |
| El Salvador | 203.9791 | 240.2641 | 173.2101 |
| Equatorial Guinea | 1207.065 | 1400.38 | 1011.316 |
| Eritrea | 1349.311 | 1574.179 | 1133.765 |
| Estonia | 333.3655 | 429.2781 | 248.4898 |
| Eswatini | 959.4604 | 1132.388 | 793.7354 |
| Ethiopia | 1515.048 | 1742.207 | 1297.98 |
| Fiji | 368.3267 | 463.811 | 286.764 |
| Finland | 211.896 | 276.2895 | 153.3139 |
| France | 237.4705 | 316.5898 | 174.8284 |
| Gabon | 943.644 | 1116.115 | 780.9278 |
| Gambia | 1382.69 | 1604.631 | 1157.654 |
| Georgia | 64.26074 | 68.56708 | 60.60119 |
| Germany | 274.0297 | 358.477 | 204.066 |
| Ghana | 587.0967 | 646.1743 | 533.0578 |
| Greece | 136.6616 | 182.5232 | 99.77468 |
| Greenland | 99.93841 | 129.8541 | 75.26033 |
| Grenada | 297.7589 | 369.6925 | 236.5641 |
| Guam | 514.9954 | 645.7307 | 408.6405 |
| Guatemala | 128.855 | 148.9345 | 112.041 |
| Guinea | 1578.59 | 1854.1 | 1330.721 |
| Guinea-Bissau | 1374.23 | 1588.984 | 1165.945 |
| Guyana | 329.4607 | 411.9199 | 260.8336 |
| Haiti | 463.0804 | 575.262 | 366.2513 |
| Honduras | 245.5393 | 287.6921 | 209.6585 |
| Hungary | 90.19708 | 115.5003 | 69.99201 |
| Iceland | 144.8756 | 190.5076 | 109.1878 |
| India | 358.3636 | 426.3861 | 298.305 |
| Indonesia | 358.8889 | 422.4764 | 303.077 |
| Iran (Islamic Republic of) | 292.574 | 363.9084 | 230.0421 |
| Iraq | 343.7393 | 437.1885 | 270.6629 |
| Ireland | 125.2824 | 164.3574 | 94.18331 |
| Israel | 299.8869 | 391.4204 | 223.0486 |
| Italy | 115.2191 | 140.2812 | 95.36137 |
| Jamaica | 246.4075 | 305.1784 | 196.8356 |
| Japan | 204.1044 | 237.7939 | 175.5159 |
| Jordan | 170.9738 | 214.3486 | 135.5446 |
| Kazakhstan | 293.2819 | 369.6199 | 226.1901 |
| Kenya | 1061.698 | 1240.025 | 890.6285 |
| Kiribati | 426.204 | 546.2419 | 326.4768 |
| Kuwait | 150.1417 | 190.4056 | 117.0146 |
| Kyrgyzstan | 155.4804 | 197.39 | 121.2222 |
| Lao People's Democratic Republic | 546.7199 | 675.0036 | 433.4563 |
| Latvia | 308.656 | 400.8283 | 229.0153 |
| Lebanon | 294.2141 | 370.7751 | 229.3877 |
| Lesotho | 826.1003 | 992.7413 | 674.9351 |
| Liberia | 1171.053 | 1370.021 | 983.1251 |
| Libya | 225.1951 | 293.6227 | 170.9474 |
| Lithuania | 352.0213 | 457.9192 | 255.4659 |
| Luxembourg | 58.27394 | 74.76855 | 45.33542 |
| Madagascar | 1317.043 | 1556.972 | 1090.778 |
| Malawi | 1364.991 | 1586.522 | 1160.107 |
| Malaysia | 522.1854 | 660.5645 | 407.5384 |
| Maldives | 437.7819 | 548.4524 | 340.1574 |
| Mali | 1928.746 | 2258.477 | 1617.892 |
| Malta | 184.7156 | 243.2489 | 137.357 |
| Marshall Islands | 353.3467 | 441.3228 | 277.8423 |
| Mauritania | 1317.215 | 1505.16 | 1125.323 |
| Mauritius | 305.3254 | 384.0425 | 236.0042 |
| Mexico | 519.5044 | 599.7773 | 446.5888 |
| Micronesia (Federated States of) | 373.8944 | 466.5984 | 291.6526 |
| Monaco | 147.2639 | 194.5564 | 107.2373 |
| Mongolia | 197.266 | 252.5979 | 154.4756 |
| Montenegro | 172.8053 | 228.523 | 126.4193 |
| Morocco | 305.8856 | 377.8355 | 242.9138 |
| Mozambique | 1601.491 | 1855.411 | 1354.223 |
| Myanmar | 483.8033 | 593.0265 | 384.1055 |
| Namibia | 979.3779 | 1172.385 | 800.241 |
| Nauru | 503.6732 | 636.2689 | 393.3868 |
| Nepal | 256.5304 | 327.9695 | 200.7278 |
| Netherlands | 102.8578 | 139.3444 | 74.8353 |
| New Zealand | 327.3468 | 382.3376 | 278.4439 |
| Nicaragua | 213.2368 | 252.4399 | 181.4007 |
| Niger | 2256.951 | 2644.811 | 1883.968 |
| Nigeria | 1731.933 | 1975.96 | 1483.116 |
| Niue | 408.8576 | 505.1421 | 319.9085 |
| North Macedonia | 138.127 | 180.4149 | 103.2777 |
| Northern Mariana Islands | 325.3121 | 419.1931 | 250.1963 |
| Norway | 380.9094 | 472.0612 | 289.8479 |
| Oman | 308.6551 | 396.8088 | 239.3159 |
| Pakistan | 693.0434 | 846.9851 | 560.0016 |
| Palau | 321.7496 | 400.8897 | 253.7556 |
| Palestine | 287.0367 | 363.0219 | 220.7373 |
| Panama | 225.0625 | 265.9201 | 190.9146 |
| Papua New Guinea | 578.712 | 725.7735 | 458.1491 |
| Paraguay | 296.7425 | 369.3401 | 236.2754 |
| Peru | 106.7727 | 127.2465 | 90.52609 |
| Philippines | 630.6462 | 756.065 | 515.6004 |
| Poland | 124.0235 | 141.5456 | 107.6349 |
| Portugal | 104.3068 | 108.0599 | 100.5842 |
| Puerto Rico | 232.918 | 296.243 | 182.7265 |
| Qatar | 277.8677 | 354.2132 | 213.9413 |
| Republic of Korea | 36.34082 | 45.72687 | 29.60774 |
| Republic of Moldova | 235.9024 | 307.3045 | 176.3234 |
| Romania | 289.8762 | 374.3864 | 219.3431 |
| Russian Federation | 393.9611 | 497.3765 | 297.7357 |
| Rwanda | 1282.404 | 1475.787 | 1075.693 |
| Saint Kitts and Nevis | 270.8791 | 342.3686 | 214.8691 |
| Saint Lucia | 221.0524 | 272.7508 | 177.9855 |
| Saint Vincent and the Grenadines | 301.8913 | 372.5344 | 242.1673 |
| Samoa | 205.0633 | 256.2934 | 161.4625 |
| San Marino | 140.8082 | 188.8636 | 101.9788 |
| Sao Tome and Principe | 1006.786 | 1159.407 | 855.9936 |
| Saudi Arabia | 310.3732 | 387.6581 | 242.7433 |
| Senegal | 1422.413 | 1639.1 | 1208.934 |
| Serbia | 106.6818 | 141.1501 | 80.66169 |
| Seychelles | 476.7939 | 585.6346 | 374.3781 |
| Sierra Leone | 1340.852 | 1568.263 | 1125.705 |
| Singapore | 73.76722 | 97.31214 | 55.51401 |
| Slovakia | 157.602 | 204.4361 | 117.3512 |
| Slovenia | 252.3636 | 331.8334 | 186.2365 |
| Solomon Islands | 570.0982 | 708.4399 | 452.4489 |
| Somalia | 2251.261 | 2597.086 | 1893.54 |
| South Africa | 740.708 | 875.867 | 617.1958 |
| South Sudan | 1909.692 | 2215.527 | 1588.825 |
| Spain | 137.5891 | 181.4137 | 102.4035 |
| Sri Lanka | 393.1184 | 495.9096 | 301.9591 |
| Sudan | 432.9985 | 546.6929 | 341.9474 |
| Suriname | 352.8531 | 439.6313 | 281.9507 |
| Sweden | 105.6604 | 134.4118 | 83.33214 |
| Switzerland | 134.423 | 179.0507 | 98.92026 |
| Syrian Arab Republic | 261.7402 | 329.9487 | 206.2617 |
| Taiwan (Province of China) | 65.22897 | 83.82221 | 51.01922 |
| Tajikistan | 182.3424 | 232.8351 | 143.5666 |
| Thailand | 259.5548 | 321.9355 | 205.291 |
| Timor-Leste | 848.9236 | 1044.362 | 668.2808 |
| Togo | 1291.635 | 1508.446 | 1065.868 |
| Tokelau | 651.5646 | 825.5428 | 505.6129 |
| Tonga | 539.2179 | 688.4055 | 406.2897 |
| Trinidad and Tobago | 262.0195 | 326.0192 | 207.9992 |
| Tunisia | 232.5643 | 299.737 | 174.0605 |
| Turkey | 287.1069 | 367.2261 | 217.0625 |
| Turkmenistan | 172.9679 | 225.2063 | 131.9801 |
| Tuvalu | 372.207 | 470.9364 | 286.4501 |
| Uganda | 1590.099 | 1872.398 | 1313.376 |
| Ukraine | 310.4417 | 397.1002 | 235.061 |
| United Arab Emirates | 176.7892 | 220.9106 | 136.115 |
| United Kingdom | 204.9758 | 261.1933 | 157.5116 |
| United Republic of Tanzania | 1537.322 | 1812.558 | 1301.143 |
| United States of America | 354.0811 | 401.9803 | 308.9449 |
| United States Virgin Islands | 349.0728 | 445.6807 | 274.6254 |
| Uruguay | 324.6534 | 414.8562 | 248.4667 |
| Uzbekistan | 138.0875 | 178.1388 | 106.5797 |
| Vanuatu | 444.3054 | 562.2129 | 352.0143 |
| Venezuela (Bolivarian Republic of) | 227.0209 | 265.8864 | 194.4228 |
| Viet Nam | 316.9648 | 402.0994 | 246.7355 |
| Yemen | 687.4017 | 865.8294 | 544.5198 |
| Zambia | 1587.76 | 1813.945 | 1349.157 |
| Zimbabwe | 1052.251 | 1277.819 | 855.235 |

ASIR, age-standardized incidence rate; HDP, hypertensive disorders of pregnancy.
