# Supplementary material for: Epidemiological trends of maternal hypertensive disorders of pregnancy at the global, regional, and national levels: a population‐based study
Source: BMC Pregnancy Childbirth. 2021 May 8;21:364. doi: 10.1186/s12884-021-03809-2 (PMC8106862; doi:10.1186/s12884-021-03809-2)
Supplement: Supplementary file 6 — Supplementary Table 4.The prevalence of HDP in different countries and regions in 2019. [file 12884_2021_3809_MOESM6_ESM.docx]

Supplementary Table 4 The prevalence of HDP at different countries and regions in 2019

| Countries and regions | Prevalence | 95% UI upper | 95% UI lower |
| --- | --- | --- | --- |
| Afghanistan | 22320.9 | 32865.93 | 13993.42 |
| Albania | 535.5114 | 822.2412 | 323.8339 |
| Algeria | 22010.99 | 32471.65 | 13480.42 |
| American Samoa | 22.17435 | 32.61917 | 13.55052 |
| Andorra | 9.96924 | 14.90894 | 5.900095 |
| Angola | 48629.07 | 69441.68 | 31098.49 |
| Antigua and Barbuda | 21.89405 | 32.37063 | 13.64846 |
| Argentina | 17557.73 | 26052.46 | 11047.59 |
| Armenia | 308.238 | 463.0666 | 191.626 |
| Australia | 5186.471 | 7816.204 | 2991.84 |
| Austria | 659.2853 | 897.0788 | 452.0526 |
| Azerbaijan | 1051.61 | 1551.134 | 657.2769 |
| Bahamas | 97.8187 | 145.8286 | 61.16358 |
| Bahrain | 349.3796 | 513.175 | 213.7209 |
| Bangladesh | 45830.73 | 67333.01 | 29167.07 |
| Barbados | 66.60486 | 97.58923 | 42.29502 |
| Belarus | 2979.201 | 4496.608 | 1796.484 |
| Belgium | 3235.073 | 4962.657 | 1936.958 |
| Belize | 171.8179 | 256.3697 | 108.1774 |
| Benin | 22447.77 | 31565.21 | 14275.69 |
| Bermuda | 14.27557 | 21.1463 | 8.738132 |
| Bhutan | 274.8441 | 408.3098 | 171.2505 |
| Bolivia (Plurinational State of) | 3672.509 | 5386.331 | 2319.769 |
| Bosnia and Herzegovina | 396.7591 | 605.0049 | 237.3063 |
| Botswana | 2289.331 | 3293.621 | 1456.983 |
| Brazil | 56667.84 | 79804.43 | 36607.82 |
| Brunei Darussalam | 53.30733 | 80.32838 | 32.43787 |
| Bulgaria | 705.716 | 989.199 | 461.9852 |
| Burkina Faso | 41502.84 | 59777.44 | 26595.99 |
| Burundi | 21540.69 | 30770.71 | 13787.46 |
| Cabo Verde | 497.2967 | 698.7101 | 318.5744 |
| Cambodia | 9365.838 | 14390.58 | 5727.125 |
| Cameroon | 41831.84 | 58790.87 | 26714.33 |
| Canada | 1353.184 | 1966.926 | 868.0573 |
| Central African Republic | 8154.144 | 11857.32 | 5159.892 |
| Chad | 32399.33 | 46656.54 | 20626.11 |
| Chile | 6444.374 | 9623.67 | 3945.923 |
| China | 131466.3 | 195408.3 | 82707.93 |
| Colombia | 6805.004 | 9980.997 | 4438.71 |
| Comoros | 820.6493 | 1150.477 | 529.357 |
| Congo | 6644.575 | 9411.649 | 4230.163 |
| Cook Islands | 6.588009 | 9.987042 | 4.070043 |
| Costa Rica | 641.9572 | 940.7065 | 415.4849 |
| Croatia | 374.0686 | 508.6267 | 259.8688 |
| Cuba | 2312.157 | 3424.652 | 1437.236 |
| Cyprus | 104.7985 | 161.8531 | 62.32677 |
| Czechia | 2304.732 | 3455.148 | 1372.756 |
| Cote d'Ivoire | 41111.05 | 57954.61 | 26557.21 |
| Democratic People's Republic of Korea | 1980.736 | 3128.889 | 1172.571 |
| Democratic Republic of the Congo | 130781.6 | 189382.7 | 81338.23 |
| Denmark | 406.5382 | 652.7397 | 243.0879 |
| Djibouti | 1765.032 | 2484.234 | 1152.573 |
| Dominica | 18.3045 | 27.3895 | 11.23867 |
| Dominican Republic | 4750.935 | 7086.402 | 2951.558 |
| Ecuador | 11759.46 | 15920.65 | 8119.42 |
| Egypt | 39930.8 | 60337.53 | 24570.22 |
| El Salvador | 965.0592 | 1402.352 | 626.2235 |
| Equatorial Guinea | 1816.155 | 2589.2 | 1149.875 |
| Eritrea | 9305.113 | 13108.09 | 6059.402 |
| Estonia | 450.8292 | 683.2111 | 265.7239 |
| Eswatini | 1381.457 | 1973.928 | 875.9981 |
| Ethiopia | 138491.9 | 196252.5 | 88582.4 |
| Fiji | 344.0969 | 513.8682 | 209.3972 |
| Finland | 1136.986 | 1699.442 | 666.5238 |
| France | 14994.08 | 22603.05 | 8842.967 |
| Gabon | 2017.224 | 2826.745 | 1291.466 |
| Gambia | 3301.502 | 4647.504 | 2143.128 |
| Georgia | 169.0978 | 227.8015 | 121.0008 |
| Germany | 21519.23 | 33566.18 | 12838.22 |
| Ghana | 12837.4 | 17305.64 | 8801.891 |
| Greece | 1316.409 | 1987.423 | 778.6339 |
| Greenland | 5.558986 | 8.600646 | 3.266175 |
| Grenada | 30.30194 | 44.80093 | 18.93153 |
| Guam | 77.97286 | 115.9899 | 48.05881 |
| Guatemala | 392.4424 | 434.8832 | 354.4932 |
| Guinea | 20551.68 | 29201.11 | 13145.89 |
| Guinea-Bissau | 2825.189 | 3952.792 | 1834.589 |
| Guyana | 284.6958 | 429.2707 | 176.6463 |
| Haiti | 6349.751 | 9414.845 | 3906.297 |
| Honduras | 1807.901 | 2680.08 | 1164.994 |
| Hungary | 728.9683 | 1102.814 | 453.4857 |
| Iceland | 50.39534 | 77.59537 | 29.17252 |
| India | 423997.4 | 623364.2 | 266164.8 |
| Indonesia | 94620.57 | 136478.3 | 59992.42 |
| Iran (Islamic Republic of) | 32901.05 | 47514.55 | 20479.38 |
| Iraq | 17636.46 | 25507.22 | 11066.64 |
| Ireland | 631.9526 | 982.6329 | 385.0762 |
| Israel | 2844.378 | 4289.282 | 1710.902 |
| Italy | 5800.448 | 8235.018 | 3697.613 |
| Jamaica | 768.3703 | 1128.103 | 476.324 |
| Japan | 16621.56 | 23592.46 | 10957.97 |
| Jordan | 1941.253 | 2867.048 | 1213.136 |
| Kazakhstan | 6539.406 | 9849.39 | 4071.551 |
| Kenya | 57340.54 | 81737.03 | 36400.03 |
| Kiribati | 54.50293 | 81.97211 | 32.26141 |
| Kuwait | 1013.14 | 1507.856 | 615.4108 |
| Kyrgyzstan | 1107.925 | 1678.905 | 684.8501 |
| Lao People's Democratic Republic | 4356.478 | 6442.299 | 2734.321 |
| Latvia | 578.9355 | 860.2257 | 343.0763 |
| Lebanon | 1807.803 | 2673.073 | 1135.821 |
| Lesotho | 2119.787 | 3061.926 | 1341.442 |
| Liberia | 5980.379 | 8440.855 | 3873.484 |
| Libya | 2055.324 | 3072.137 | 1261.414 |
| Lithuania | 989.4837 | 1501.168 | 595.1619 |
| Luxembourg | 33.12456 | 52.89061 | 19.91824 |
| Madagascar | 36794.44 | 52437.61 | 23619.33 |
| Malawi | 26107.64 | 37285.46 | 17093.24 |
| Malaysia | 19104.37 | 28883.68 | 11692.12 |
| Maldives | 232.3114 | 345.0802 | 138.4531 |
| Mali | 40366.46 | 57712.8 | 25907.07 |
| Malta | 82.25888 | 126.1726 | 48.52256 |
| Marshall Islands | 21.061 | 30.75714 | 12.75309 |
| Mauritania | 5216.232 | 7349.34 | 3391.615 |
| Mauritius | 399.1905 | 604.5707 | 245.9186 |
| Mexico | 65584.17 | 92543.37 | 42766.05 |
| Micronesia (Federated States of) | 36.61699 | 55.13161 | 22.58421 |
| Monaco | 4.332552 | 6.603224 | 2.545083 |
| Mongolia | 784.1135 | 1195.073 | 480.2763 |
| Montenegro | 107.3604 | 166.5124 | 63.74653 |
| Morocco | 12058.26 | 17700.86 | 7505.075 |
| Mozambique | 48557.82 | 68942.35 | 31196.58 |
| Myanmar | 29221.27 | 43336.81 | 17843.88 |
| Namibia | 2834.789 | 4093.572 | 1769.489 |
| Nauru | 5.761585 | 8.613639 | 3.513251 |
| Nepal | 10389.41 | 15396.63 | 6602.871 |
| Netherlands | 1567.472 | 2481.692 | 915.9828 |
| New Zealand | 1448.882 | 2024.506 | 936.3394 |
| Nicaragua | 1057.04 | 1565.306 | 676.2081 |
| Niger | 46631.04 | 66440.61 | 29334.67 |
| Nigeria | 367632.3 | 518038.4 | 234623.3 |
| Niue | 0.596312 | 0.887861 | 0.362303 |
| North Macedonia | 317.2402 | 489.1806 | 187.3597 |
| Northern Mariana Islands | 10.4057 | 15.58655 | 6.538331 |
| Norway | 2045.163 | 3050.628 | 1220.49 |
| Oman | 1493.984 | 2221.849 | 920.8578 |
| Pakistan | 143309.6 | 209804.7 | 89458.08 |
| Palau | 4.458325 | 6.506623 | 2.837299 |
| Palestine | 1700.534 | 2566.775 | 1073.181 |
| Panama | 637.2972 | 931.3776 | 402.3092 |
| Papua New Guinea | 5654.289 | 8450.591 | 3402.508 |
| Paraguay | 2093.651 | 3155.353 | 1305.684 |
| Peru | 2624.871 | 3910.535 | 1631.011 |
| Philippines | 69853.67 | 101456.8 | 43908.12 |
| Poland | 4607.999 | 6399.742 | 3090.426 |
| Portugal | 1068.872 | 1467.76 | 738.571 |
| Puerto Rico | 705.0885 | 1071.267 | 438.2622 |
| Qatar | 676.6848 | 1016.547 | 396.4649 |
| Republic of Korea | 819.135 | 1363.402 | 445.9108 |
| Republic of Moldova | 946.2573 | 1413.705 | 583.5905 |
| Romania | 4956.453 | 7377.562 | 2969.232 |
| Russian Federation | 60180.76 | 89929.66 | 37125.23 |
| Rwanda | 17741.53 | 25216.34 | 11609.41 |
| Saint Kitts and Nevis | 16.27497 | 24.39298 | 10.1713 |
| Saint Lucia | 39.18654 | 57.53083 | 25.22505 |
| Saint Vincent and the Grenadines | 32.87743 | 49.07567 | 20.49589 |
| Samoa | 38.12673 | 56.70369 | 23.00798 |
| San Marino | 4.731348 | 7.378488 | 2.746147 |
| Sao Tome and Principe | 218.8556 | 309.2419 | 142.5987 |
| Saudi Arabia | 14813.9 | 21762.48 | 9044.247 |
| Senegal | 21244.23 | 30086.88 | 13748.29 |
| Serbia | 853.4998 | 1336.386 | 519.9083 |
| Seychelles | 46.57264 | 68.36231 | 29.06739 |
| Sierra Leone | 12172.59 | 17573.18 | 7832.882 |
| Singapore | 551.2682 | 837.8423 | 330.3018 |
| Slovakia | 865.3879 | 1331.072 | 518.4443 |
| Slovenia | 484.8461 | 740.5224 | 285.331 |
| Solomon Islands | 366.6375 | 536.1343 | 225.9569 |
| Somalia | 38686.66 | 54069.98 | 24992.19 |
| South Africa | 49030.19 | 70088.64 | 30785.72 |
| South Sudan | 15933.9 | 22043.98 | 10346.68 |
| Spain | 6234.295 | 9476.79 | 3743.778 |
| Sri Lanka | 9129.622 | 13641.01 | 5497.924 |
| Sudan | 20687.68 | 30612.68 | 12897.16 |
| Suriname | 198.8708 | 297.4838 | 124.9745 |
| Sweden | 1060.055 | 1551.026 | 656.4035 |
| Switzerland | 1232.722 | 1891.101 | 713.8277 |
| Syrian Arab Republic | 4248.497 | 6155.117 | 2691.049 |
| Taiwan (Province of China) | 1267.627 | 1993.522 | 740.2154 |
| Tajikistan | 1894.172 | 2909.536 | 1165.189 |
| Thailand | 17762.98 | 26468.2 | 11229.12 |
| Timor-Leste | 1028.314 | 1530.412 | 638.8404 |
| Togo | 9837.544 | 14551.89 | 6236.348 |
| Tokelau | 0.808375 | 1.206725 | 0.483786 |
| Tonga | 52.48267 | 78.45967 | 32.66758 |
| Trinidad and Tobago | 357.6008 | 536.2816 | 215.5423 |
| Tunisia | 3385.018 | 4950.727 | 2084.374 |
| Turkey | 30047.54 | 44844.72 | 17986.72 |
| Turkmenistan | 903.3857 | 1407.82 | 534.6345 |
| Tuvalu | 4.142084 | 6.235643 | 2.504688 |
| Uganda | 69473.62 | 98873.96 | 44349.91 |
| Ukraine | 13818.18 | 20594.6 | 8366.291 |
| United Arab Emirates | 1545.829 | 2281.872 | 949.9247 |
| United Kingdom | 14082.49 | 20723.26 | 8445.741 |
| United Republic of Tanzania | 93778.44 | 132698.5 | 60964.92 |
| United States of America | 112074.3 | 155942.6 | 73012.55 |
| United States Virgin Islands | 29.46521 | 43.9605 | 17.91049 |
| Uruguay | 1206.511 | 1762.272 | 748.7755 |
| Uzbekistan | 5206.435 | 7875.991 | 3140.522 |
| Vanuatu | 130.6439 | 193.8246 | 80.37412 |
| Venezuela (Bolivarian Republic of) | 4272.812 | 6244.356 | 2728.018 |
| Viet Nam | 35350.13 | 53780.11 | 21334 |
| Yemen | 25222.17 | 37269.78 | 15796.46 |
| Zambia | 28709.22 | 40805.56 | 17952.61 |
| Zimbabwe | 19115.78 | 27656.69 | 11836.04 |

HDP, hypertensive disorders of pregnancy.
