# Supplementary material for: Epidemiological trends of maternal hypertensive disorders of pregnancy at the global, regional, and national levels: a population‐based study
Source: BMC Pregnancy Childbirth. 2021 May 8;21:364. doi: 10.1186/s12884-021-03809-2 (PMC8106862; doi:10.1186/s12884-021-03809-2)
Supplement: Supplementary file 7 — Supplementary Table 5.The EACP of the ASIR of HDP in different countries and regions from 1990 to 2019. [file 12884_2021_3809_MOESM7_ESM.docx]

Supplementary Table 5 The EACP of ASIR of HDP at different countries and regions during 1990 to 2019

| Countries and regions | EACP of ASIR | 95% UI upper | 95% UI lower |
| --- | --- | --- | --- |
| Afghanistan | -1.042114353 | -0.88791 | -1.19608 |
| Albania | -1.512975007 | -1.23055 | -1.79459 |
| Algeria | -0.483075246 | -0.27594 | -0.68978 |
| American Samoa | -1.225006504 | -1.04974 | -1.39996 |
| Andorra | 0.269649075 | 0.6569 | -0.11611 |
| Angola | -1.119098821 | -1.0268 | -1.21131 |
| Antigua and Barbuda | -1.506037718 | -1.26744 | -1.74406 |
| Argentina | -0.407992579 | -0.19145 | -0.62407 |
| Armenia | -0.310682342 | 0.119057 | -0.73858 |
| Australia | -1.137745538 | -0.84397 | -1.43065 |
| Austria | -0.539158774 | -0.10565 | -0.97079 |
| Azerbaijan | -0.777371892 | -0.38418 | -1.16901 |
| Bahamas | -1.937083469 | -1.71233 | -2.16133 |
| Bahrain | -2.442912242 | -2.21591 | -2.66938 |
| Bangladesh | -3.055719005 | -2.85992 | -3.25113 |
| Barbados | -0.919426978 | -0.68239 | -1.1559 |
| Belarus | 0.17120627 | 0.437314 | -0.0942 |
| Belgium | 0.120178702 | 0.38701 | -0.14594 |
| Belize | -2.218880378 | -2.04025 | -2.39718 |
| Benin | -0.245524858 | -0.15457 | -0.33639 |
| Bermuda | -0.676578495 | -0.44167 | -0.91093 |
| Bhutan | -3.160407503 | -2.97575 | -3.34471 |
| Bolivia (Plurinational State of) | -1.376199139 | -1.16865 | -1.58331 |
| Bosnia and Herzegovina | -0.751952668 | -0.38611 | -1.11646 |
| Botswana | -1.47423981 | -1.34023 | -1.60807 |
| Brazil | -1.487519816 | -1.25467 | -1.71982 |
| Brunei Darussalam | -1.879349657 | -1.55024 | -2.20736 |
| Bulgaria | 0.100090183 | 0.482375 | -0.28074 |
| Burkina Faso | -0.966581939 | -0.87814 | -1.05494 |
| Burundi | -0.691820908 | -0.60416 | -0.77941 |
| Cabo Verde | -2.35273737 | -2.23273 | -2.47259 |
| Cambodia | -3.324651918 | -3.17434 | -3.47473 |
| Cameroon | -1.119051945 | -1.02204 | -1.21597 |
| Canada | -1.079158694 | -0.7153 | -1.44168 |
| Central African Republic | -0.978284232 | -0.88036 | -1.07611 |
| Chad | -0.344294706 | -0.26106 | -0.42746 |
| Chile | -1.041866418 | -0.79728 | -1.28585 |
| China | -1.497201725 | -1.10882 | -1.88406 |
| Colombia | -1.564154106 | -1.30868 | -1.81896 |
| Comoros | -2.404326175 | -2.30211 | -2.50644 |
| Congo | -1.198560766 | -1.09166 | -1.30535 |
| Cook Islands | -0.854023925 | -0.66118 | -1.04649 |
| Costa Rica | -1.7824287 | -1.50446 | -2.05962 |
| Cote d'Ivoire | -1.11799502 | -1.02032 | -1.21557 |
| Croatia | 1.050080229 | 1.491877 | 0.610206 |
| Cuba | 0.106042874 | 0.364853 | -0.1521 |
| Cyprus | -3.045819981 | -2.59975 | -3.48984 |
| Czechia | 0.461066197 | 0.779604 | 0.143536 |
| Democratic People's Republic of Korea | -3.020525307 | -2.66843 | -3.37134 |
| Democratic Republic of the Congo | -1.185886078 | -1.09473 | -1.27696 |
| Denmark | 1.521754643 | 2.063003 | 0.983377 |
| Djibouti | -1.807825185 | -1.71559 | -1.89997 |
| Dominica | -1.98423751 | -1.77111 | -2.19691 |
| Dominican Republic | -1.247959168 | -1.05717 | -1.43838 |
| Ecuador | 2.093430649 | 2.28469 | 1.902529 |
| Egypt | -1.972658344 | -1.78991 | -2.15506 |
| El Salvador | -2.118873403 | -1.864 | -2.37308 |
| Equatorial Guinea | -2.339445582 | -2.24448 | -2.43431 |
| Eritrea | -1.699623192 | -1.60398 | -1.79517 |
| Estonia | 0.777360446 | 1.01795 | 0.537344 |
| Eswatini | -1.818650529 | -1.70438 | -1.93279 |
| Ethiopia | -1.505765243 | -1.41441 | -1.59703 |
| Fiji | 0.049571709 | 0.263173 | -0.16357 |
| Finland | -1.19711153 | -0.91218 | -1.48122 |
| France | 0.367176202 | 0.652335 | 0.082826 |
| Gabon | -2.19856573 | -2.08552 | -2.31148 |
| Gambia | -1.633020611 | -1.53833 | -1.72762 |
| Georgia | 1.148386806 | 1.698239 | 0.601508 |
| Germany | 0.247597222 | 0.522961 | -0.02701 |
| Ghana | -1.770837955 | -1.66773 | -1.87384 |
| Greece | 0.937738989 | 1.308865 | 0.567973 |
| Greenland | -0.423800393 | -0.02819 | -0.81785 |
| Grenada | -1.781943006 | -1.56717 | -1.99625 |
| Guam | 0.181066675 | 0.366578 | -0.0041 |
| Guatemala | -3.15694587 | -2.85728 | -3.45569 |
| Guinea | -1.301334762 | -1.20778 | -1.3948 |
| Guinea-Bissau | -1.437041571 | -1.34002 | -1.53396 |
| Guyana | -1.137334493 | -0.93176 | -1.34248 |
| Haiti | -2.000131767 | -1.83565 | -2.16434 |
| Honduras | -2.681874231 | -2.45812 | -2.90512 |
| Hungary | -0.27767134 | 0.180464 | -0.73371 |
| Iceland | -0.972386373 | -0.61197 | -1.33149 |
| India | -2.829794381 | -2.66053 | -2.99877 |
| Indonesia | -1.694176254 | -1.50656 | -1.88144 |
| Iran (Islamic Republic of) | -1.633596678 | -1.4119 | -1.85479 |
| Iraq | -2.924919412 | -2.74862 | -3.1009 |
| Ireland | 0.107165138 | 0.487957 | -0.27218 |
| Israel | -0.28477558 | -0.02081 | -0.54804 |
| Italy | -0.930972658 | -0.56211 | -1.29847 |
| Jamaica | -2.020423114 | -1.7966 | -2.24374 |
| Japan | -1.653084156 | -1.37965 | -1.92576 |
| Jordan | -1.571216994 | -1.28031 | -1.86126 |
| Kazakhstan | 1.339093219 | 1.606569 | 1.072322 |
| Kenya | -2.205289145 | -2.09868 | -2.31178 |
| Kiribati | -0.835785972 | -0.65129 | -1.01994 |
| Kuwait | -2.878733342 | -2.64765 | -3.10927 |
| Kyrgyzstan | 0.175078852 | 0.506742 | -0.15549 |
| Lao People's Democratic Republic | -2.687934831 | -2.5435 | -2.83215 |
| Latvia | -0.613883218 | -0.33715 | -0.88985 |
| Lebanon | -2.261880325 | -2.0594 | -2.46394 |
| Lesotho | -1.815121082 | -1.68977 | -1.94031 |
| Liberia | -1.994537574 | -1.8952 | -2.09377 |
| Libya | -3.330009364 | -3.11519 | -3.54436 |
| Lithuania | -2.619477916 | -2.37952 | -2.85885 |
| Luxembourg | -0.24002188 | 0.357148 | -0.83364 |
| Madagascar | -1.466753448 | -1.36812 | -1.56529 |
| Malawi | -1.767788698 | -1.67435 | -1.86114 |
| Malaysia | -1.513413417 | -1.34611 | -1.68044 |
| Maldives | -2.275964593 | -2.10049 | -2.45112 |
| Mali | -0.663085423 | -0.57428 | -0.75182 |
| Malta | -1.952326276 | -1.6381 | -2.26555 |
| Marshall Islands | -1.043895106 | -0.84073 | -1.24664 |
| Mauritania | -1.698672078 | -1.60277 | -1.79448 |
| Mauritius | -1.203836344 | -0.98692 | -1.42028 |
| Mexico | -1.205021077 | -1.02608 | -1.38364 |
| Micronesia (Federated States of) | -2.220827949 | -2.03897 | -2.40235 |
| Monaco | 0.047947457 | 0.390675 | -0.29361 |
| Mongolia | 0.174629833 | 0.484657 | -0.13444 |
| Montenegro | -0.382049894 | -0.07021 | -0.69291 |
| Morocco | -2.023980857 | -1.8247 | -2.22286 |
| Mozambique | -0.751176292 | -0.65594 | -0.84632 |
| Myanmar | -1.679705027 | -1.5143 | -1.84484 |
| Namibia | -1.251510039 | -1.13119 | -1.37168 |
| Nauru | -1.580248606 | -1.41547 | -1.74475 |
| Nepal | -4.643414581 | -4.45485 | -4.83161 |
| Netherlands | 0.540707566 | 0.984685 | 0.098682 |
| New Zealand | -1.256154282 | -1.06061 | -1.45132 |
| Nicaragua | -2.377334242 | -2.13038 | -2.62366 |
| Niger | -0.172335373 | -0.08748 | -0.25712 |
| Nigeria | -0.939648 | -0.85064 | -1.02858 |
| Niue | -0.987738394 | -0.7991 | -1.17602 |
| North Macedonia | -1.205507541 | -0.87764 | -1.53229 |
| Northern Mariana Islands | -0.245659471 | -0.02378 | -0.46705 |
| Norway | -1.463510357 | -1.2278 | -1.69866 |
| Oman | -3.682745263 | -3.50033 | -3.86482 |
| Pakistan | -2.227803757 | -2.09787 | -2.35757 |
| Palau | -0.754426762 | -0.53708 | -0.9713 |
| Palestine | -3.519082172 | -3.32452 | -3.71325 |
| Panama | -0.340926083 | -0.07022 | -0.6109 |
| Papua New Guinea | -0.468489361 | -0.30306 | -0.63364 |
| Paraguay | -1.713026906 | -1.50086 | -1.92474 |
| Peru | -1.690777136 | -1.40806 | -1.97269 |
| Philippines | -1.898270587 | -1.75782 | -2.03852 |
| Poland | -1.84579558 | -1.50738 | -2.18305 |
| Portugal | -0.515303379 | -0.0987 | -0.93017 |
| Puerto Rico | -1.978140411 | -1.75864 | -2.19715 |
| Qatar | -3.969878944 | -3.75071 | -4.18855 |
| Republic of Korea | -2.377019158 | -1.6825 | -3.06663 |
| Republic of Moldova | -1.406096145 | -1.15498 | -1.65658 |
| Romania | 0.276248475 | 0.556037 | -0.00276 |
| Russian Federation | 1.256438788 | 1.490394 | 1.023023 |
| Rwanda | -2.438387023 | -2.34297 | -2.53371 |
| Saint Kitts and Nevis | -1.644169428 | -1.42394 | -1.8639 |
| Saint Lucia | -2.743277891 | -2.5129 | -2.97311 |
| Saint Vincent and the Grenadines | -1.216832802 | -1.00009 | -1.4331 |
| Samoa | -1.981396223 | -1.7296 | -2.23255 |
| San Marino | 0.282101172 | 0.638247 | -0.07278 |
| Sao Tome and Principe | -2.21759967 | -2.11275 | -2.32234 |
| Saudi Arabia | -3.207499139 | -3.02298 | -3.39166 |
| Senegal | -1.48013163 | -1.38345 | -1.57672 |
| Serbia | -1.584328482 | -1.17291 | -1.99404 |
| Seychelles | -0.029611899 | 0.162876 | -0.22173 |
| Sierra Leone | -1.248353351 | -1.14908 | -1.34753 |
| Singapore | -0.938124273 | -0.49177 | -1.38248 |
| Slovakia | -0.116192821 | 0.267166 | -0.49809 |
| Slovenia | 0.283314813 | 0.580721 | -0.01321 |
| Solomon Islands | -1.328164075 | -1.17462 | -1.48147 |
| Somalia | -0.543778796 | -0.46125 | -0.62624 |
| South Africa | -0.950330126 | -0.81007 | -1.09039 |
| South Sudan | -0.418567804 | -0.32823 | -0.50883 |
| Spain | 0.833922456 | 1.202608 | 0.46658 |
| Sri Lanka | -0.351371977 | -0.15587 | -0.54649 |
| Sudan | -2.324444475 | -2.1613 | -2.48732 |
| Suriname | -0.884033861 | -0.67948 | -1.08817 |
| Sweden | -1.225372881 | -0.86296 | -1.58646 |
| Switzerland | 1.551759019 | 1.957608 | 1.147526 |
| Syrian Arab Republic | -2.513172309 | -2.31432 | -2.71162 |
| Taiwan (Province of China) | -0.431349705 | 0.100743 | -0.96061 |
| Tajikistan | -1.256433585 | -0.96637 | -1.54564 |
| Thailand | -1.180886017 | -0.94958 | -1.41166 |
| Timor-Leste | -1.718563704 | -1.59908 | -1.83791 |
| Togo | -0.570779397 | -0.47309 | -0.66837 |
| Tokelau | -0.796492972 | -0.64397 | -0.94878 |
| Tonga | -0.417424973 | -0.25026 | -0.58431 |
| Trinidad and Tobago | -0.680017685 | -0.43796 | -0.92149 |
| Tunisia | -1.939820366 | -1.70327 | -2.17581 |
| Turkey | -0.67008699 | -0.47313 | -0.86666 |
| Turkmenistan | -1.120711873 | -0.81955 | -1.42096 |
| Tuvalu | -1.191170663 | -0.99644 | -1.38551 |
| Uganda | -1.272431739 | -1.18178 | -1.363 |
| Ukraine | 0.056842099 | 0.297304 | -0.18304 |
| United Arab Emirates | -1.792144135 | -1.53451 | -2.0491 |
| United Kingdom | 0.475198527 | 0.770335 | 0.180927 |
| United Republic of Tanzania | -0.59971165 | -0.50654 | -0.69279 |
| United States Virgin Islands | -1.690373067 | -1.50221 | -1.87817 |
| United States of America | -0.379184217 | -0.16693 | -0.59099 |
| Uruguay | -0.308976424 | -0.08322 | -0.53422 |
| Uzbekistan | -1.559301935 | -1.22595 | -1.89153 |
| Vanuatu | -1.492144439 | -1.31694 | -1.66704 |
| Venezuela (Bolivarian Republic of) | -1.126467565 | -0.8758 | -1.3765 |
| Viet Nam | -1.820840439 | -1.61843 | -2.02284 |
| Yemen | -2.726943715 | -2.60413 | -2.8496 |
| Zambia | -1.502557694 | -1.41306 | -1.59197 |
| Zimbabwe | -0.991401158 | -0.87495 | -1.10771 |

ASIR, age-standardized incidence rate; EAPC, estimated annual percentage change; HDP, hypertensive disorders of pregnancy.
