# Supplementary material for: Epidemiological trends of maternal hypertensive disorders of pregnancy at the global, regional, and national levels: a population‐based study
Source: BMC Pregnancy Childbirth. 2021 May 8;21:364. doi: 10.1186/s12884-021-03809-2 (PMC8106862; doi:10.1186/s12884-021-03809-2)
Supplement: Supplementary file 8 — Supplementary Table 6. The relationship between the ASIR and SDI. [file 12884_2021_3809_MOESM8_ESM.docx]

Supplementary Table 6 The relationship between ASIR and SDI

| Countries and regions | SDI | ASIR |
| --- | --- | --- |
| Chad | 0.238 | 2278.252 |
| Niger | 0.162 | 2256.951 |
| Somalia | 0.081 | 2251.261 |
| Burundi | 0.284 | 1929.532 |
| Mali | 0.263 | 1928.746 |
| Benin | 0.352 | 1926.304 |
| South Sudan | 0.363 | 1909.692 |
| Burkina Faso | 0.257 | 1884.186 |
| Nigeria | 0.515 | 1731.933 |
| Mozambique | 0.307 | 1601.491 |
| Uganda | 0.404 | 1590.099 |
| Angola | 0.47 | 1589.578 |
| Zambia | 0.505 | 1587.76 |
| Guinea | 0.325 | 1578.59 |
| Democratic Republic of the Congo | 0.382 | 1542.653 |
| United Republic of Tanzania | 0.423 | 1537.322 |
| Ethiopia | 0.343 | 1515.048 |
| Cote d'Ivoire | 0.408 | 1473.049 |
| Central African Republic | 0.274 | 1458.542 |
| Senegal | 0.389 | 1422.413 |
| Djibouti | 0.459 | 1395.901 |
| Gambia | 0.399 | 1382.69 |
| Guinea-Bissau | 0.355 | 1374.23 |
| Malawi | 0.384 | 1364.991 |
| Cameroon | 0.49 | 1358.188 |
| Eritrea | 0.396 | 1349.311 |
| Sierra Leone | 0.347 | 1340.852 |
| Mauritania | 0.496 | 1317.215 |
| Madagascar | 0.396 | 1317.043 |
| Togo | 0.417 | 1291.635 |
| Rwanda | 0.429 | 1282.404 |
| Equatorial Guinea | 0.685 | 1207.065 |
| Liberia | 0.37 | 1171.053 |
| Congo | 0.568 | 1133.299 |
| Comoros | 0.455 | 1080.661 |
| Kenya | 0.508 | 1061.698 |
| Zimbabwe | 0.476 | 1052.251 |
| Sao Tome and Principe | 0.502 | 1006.786 |
| Namibia | 0.612 | 979.3779 |
| Eswatini | 0.577 | 959.4604 |
| Gabon | 0.656 | 943.644 |
| Timor-Leste | 0.514 | 848.9236 |
| Lesotho | 0.507 | 826.1003 |
| Botswana | 0.634 | 775.7431 |
| Cabo Verde | 0.525 | 775.2664 |
| South Africa | 0.678 | 740.708 |
| Pakistan | 0.449 | 693.0434 |
| Yemen | 0.412 | 687.4017 |
| Tokelau | 0.626 | 651.5646 |
| Philippines | 0.623 | 630.6462 |
| Ecuador | 0.64 | 615.1275 |
| Ghana | 0.557 | 587.0967 |
| Afghanistan | 0.343 | 584.4843 |
| Papua New Guinea | 0.394 | 578.712 |
| Solomon Islands | 0.407 | 570.0982 |
| Lao People's Democratic Republic | 0.49 | 546.7199 |
| Tonga | 0.636 | 539.2179 |
| Malaysia | 0.737 | 522.1854 |
| Mexico | 0.649 | 519.5044 |
| Guam | 0.813 | 514.9954 |
| Nauru | 0.618 | 503.6732 |
| Cambodia | 0.469 | 494.8463 |
| Myanmar | 0.521 | 483.8033 |
| Seychelles | 0.724 | 476.7939 |
| Haiti | 0.432 | 463.0804 |
| Vanuatu | 0.485 | 444.3054 |
| Maldives | 0.562 | 437.7819 |
| American Samoa | 0.712 | 436.4702 |
| Sudan | 0.515 | 432.9985 |
| Kiribati | 0.527 | 426.204 |
| Algeria | 0.652 | 411.3353 |
| Niue | 0.711 | 408.8576 |
| Dominican Republic | 0.592 | 400.2348 |
| Cook Islands | 0.764 | 396.5173 |
| Russian Federation | 0.805 | 393.9611 |
| Sri Lanka | 0.69 | 393.1184 |
| Norway | 0.913 | 380.9094 |
| Micronesia (Federated States of) | 0.58 | 373.8944 |
| Tuvalu | 0.589 | 372.207 |
| Fiji | 0.664 | 368.3267 |
| Egypt | 0.658 | 366.6369 |
| Belize | 0.603 | 365.1282 |
| Indonesia | 0.66 | 358.8889 |
| India | 0.566 | 358.3636 |
| United States of America | 0.859 | 354.0811 |
| Marshall Islands | 0.544 | 353.3467 |
| Suriname | 0.636 | 352.8531 |
| Lithuania | 0.843 | 352.0213 |
| United States Virgin Islands | 0.799 | 349.0728 |
| Iraq | 0.671 | 343.7393 |
| Estonia | 0.835 | 333.3655 |
| Bolivia (Plurinational State of) | 0.566 | 330.763 |
| Guyana | 0.618 | 329.4607 |
| Argentina | 0.708 | 329.2512 |
| New Zealand | 0.84 | 327.3468 |
| Northern Mariana Islands | 0.771 | 325.3121 |
| Uruguay | 0.697 | 324.6534 |
| Palau | 0.738 | 321.7496 |
| Viet Nam | 0.617 | 316.9648 |
| Bhutan | 0.455 | 311.656 |
| Ukraine | 0.736 | 310.4417 |
| Saudi Arabia | 0.805 | 310.3732 |
| Latvia | 0.82 | 308.656 |
| Oman | 0.783 | 308.6551 |
| Morocco | 0.548 | 305.8856 |
| Belarus | 0.745 | 305.4828 |
| Mauritius | 0.705 | 305.3254 |
| Chile | 0.759 | 304.7571 |
| Saint Vincent and the Grenadines | 0.627 | 301.8913 |
| Israel | 0.803 | 299.8869 |
| Grenada | 0.669 | 297.7589 |
| Paraguay | 0.638 | 296.7425 |
| Lebanon | 0.708 | 294.2141 |
| Kazakhstan | 0.723 | 293.2819 |
| Iran (Islamic Republic of) | 0.67 | 292.574 |
| Romania | 0.76 | 289.8762 |
| Turkey | 0.748 | 287.1069 |
| Palestine | 0.588 | 287.0367 |
| Bangladesh | 0.483 | 280.8735 |
| Dominica | 0.729 | 278.7147 |
| Qatar | 0.83 | 277.8677 |
| Belgium | 0.851 | 277.3669 |
| Brazil | 0.64 | 276.5729 |
| Germany | 0.898 | 274.0297 |
| Saint Kitts and Nevis | 0.746 | 270.8791 |
| Trinidad and Tobago | 0.757 | 262.0195 |
| Syrian Arab Republic | 0.619 | 261.7402 |
| Thailand | 0.687 | 259.5548 |
| Nepal | 0.422 | 256.5304 |
| Slovenia | 0.84 | 252.3636 |
| Barbados | 0.742 | 250.1805 |
| Bermuda | 0.813 | 249.9429 |
| Jamaica | 0.684 | 246.4075 |
| Cuba | 0.668 | 246.3622 |
| Honduras | 0.496 | 245.5393 |
| Bahamas | 0.796 | 241.3242 |
| France | 0.834 | 237.4705 |
| Antigua and Barbuda | 0.743 | 236.2492 |
| Republic of Moldova | 0.696 | 235.9024 |
| Bahrain | 0.751 | 234.5714 |
| Puerto Rico | 0.814 | 232.918 |
| Tunisia | 0.672 | 232.5643 |
| Czechia | 0.828 | 227.6891 |
| Venezuela (Bolivarian Republic of) | 0.607 | 227.0209 |
| Libya | 0.709 | 225.1951 |
| Panama | 0.686 | 225.0625 |
| Saint Lucia | 0.67 | 221.0524 |
| Nicaragua | 0.517 | 213.2368 |
| Finland | 0.856 | 211.896 |
| Colombia | 0.633 | 206.6408 |
| Samoa | 0.641 | 205.0633 |
| United Kingdom | 0.847 | 204.9758 |
| Japan | 0.87 | 204.1044 |
| El Salvador | 0.573 | 203.9791 |
| Australia | 0.839 | 198.5863 |
| Mongolia | 0.606 | 197.266 |
| Albania | 0.681 | 190.2419 |
| Malta | 0.801 | 184.7156 |
| Tajikistan | 0.539 | 182.3424 |
| Costa Rica | 0.68 | 178.6249 |
| United Arab Emirates | 0.88 | 176.7892 |
| Turkmenistan | 0.67 | 172.9679 |
| Montenegro | 0.791 | 172.8053 |
| Jordan | 0.731 | 170.9738 |
| Slovakia | 0.812 | 157.602 |
| Kyrgyzstan | 0.596 | 155.4804 |
| Kuwait | 0.851 | 150.1417 |
| Monaco | 0.902 | 147.2639 |
| Iceland | 0.869 | 144.8756 |
| San Marino | 0.884 | 140.8082 |
| North Macedonia | 0.744 | 138.127 |
| Uzbekistan | 0.631 | 138.0875 |
| Spain | 0.767 | 137.5891 |
| Greece | 0.794 | 136.6616 |
| Switzerland | 0.929 | 134.423 |
| Guatemala | 0.526 | 128.855 |
| Bosnia and Herzegovina | 0.718 | 127.2031 |
| Ireland | 0.867 | 125.2824 |
| Bulgaria | 0.764 | 124.6706 |
| Poland | 0.802 | 124.0235 |
| Brunei Darussalam | 0.823 | 121.6909 |
| Andorra | 0.894 | 116.427 |
| Italy | 0.801 | 115.2191 |
| Peru | 0.648 | 106.7727 |
| Serbia | 0.767 | 106.6818 |
| Sweden | 0.872 | 105.6604 |
| Portugal | 0.743 | 104.3068 |
| Netherlands | 0.883 | 102.8578 |
| Croatia | 0.794 | 101.0737 |
| Greenland | 0.761 | 99.93841 |
| China | 0.686 | 98.7178 |
| Armenia | 0.689 | 97.97534 |
| Azerbaijan | 0.683 | 97.48303 |
| Hungary | 0.791 | 90.19708 |
| Democratic People's Republic of Korea | 0.558 | 88.52014 |
| Denmark | 0.89 | 84.63659 |
| Austria | 0.849 | 73.82165 |
| Singapore | 0.861 | 73.76722 |
| Taiwan (Province of China) | 0.868 | 65.22897 |
| Georgia | 0.702 | 64.26074 |
| Cyprus | 0.841 | 63.98918 |
| Luxembourg | 0.895 | 58.27394 |
| Canada | 0.873 | 49.13088 |
| Republic of Korea | 0.878 | 36.34082 |

ASIR, age-standardized incidence rate; SDI, socio-demographic index.
